# Two-Dimensional Asymmetric Multiferroics: Unique Way toward Strong Magnetoelectric Coupling and Multistate Memory

**DOI:** 10.1021/acs.jpclett.3c03527

**Published:** 2024-02-08

**Authors:** Zhichao Yu, Haoyun Bai, Bowen Li, Lun Li, Hui Pan

**Affiliations:** † Institute of Applied Physics and Materials Engineering, University of Macau, Macao SAR 999708, P. R. China; ‡ 2027 Laboratory, Tianfu Xinglong Lake Laboratory, Chengdu, Sichuan 610000, P. R. China; § Department of Physics and Chemistry, Faculty of Science and Technology, University of Macau, Macao SAR 999078, P. R. China

## Abstract

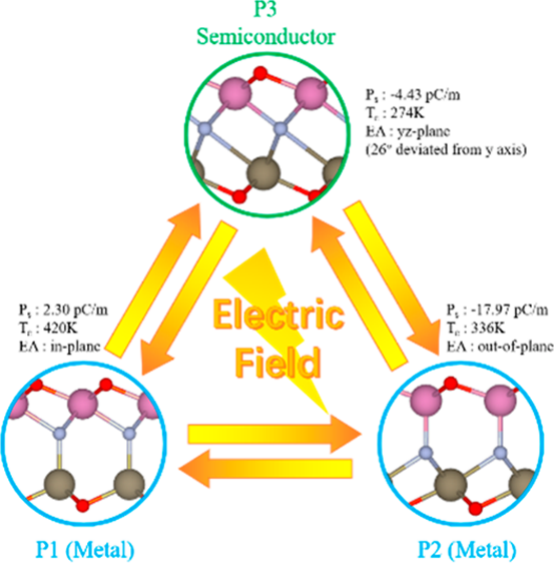

Two-dimensional (2D) materials have provided a fascinating platform for exploring novel multiferroics and emergent magnetoelectric coupling mechanisms. Here, a novel 2D asymmetric multiferroic based on Janus 2D multiferroic MXene-analogous oxynitrides (InTlNO_2_) is presented by using first-principles calculations. We find three inequivalent phases for InTlNO_2_, including two metallic phases (p1 and p2) and one semiconducting phase (p3) with a band gap of 0.88 eV. All phases are room-temperature multiferroics with different Curie temperatures, leading to tunability by phase transitions. We show that there is a 90° rotation of the magnetic anisotropy easy axis between p1 and p2, where p1 favors the in-plane and p2 the out-of-plane easy axis. Therefore, the magnetic anisotropy can be tuned by reversing the out-of-plane polarization. Our strategy provides a unique way toward strong magnetoelectric coupling and multistate memory.

Magnetoelectric coupling is greatly important for data storage, where long-sought electrically written and magnetically read data access can be achieved.^1,2^ Multiferroics in which magnetism and ferroelectricity coexist hold great potential for nonvolatile memory devices.^3−5^ According to different microscopic origins of ferroelectricity, multiferroics can be classified into two categories (types I and II).^6^ Type I multiferroics usually exhibit weak magnetoelectric coupling due to independent origins of magnetism and ferroelectricity, while type II multiferroics show weak electric polarization and low Curie temperatures (*T*_C_) because their ferroelectricity originates from magnetic spirals.^7^ Despite the advances in multiferroics both theoretically and experimentally,^8−14^ new concepts are needed for strong magnetoelectric coupling with large magnetic moments and electric polarization.

Typically, ferroelectricity is induced by spontaneous polarization, which breaks the inversion symmetry of the original structure.^15^ However, there are questions about what will happen if the inversion symmetry of the nonpolar structure is already broken by some modulations, such as heterostructure engineering and elemental substitution. Previous studies of two-dimensional (2D) multiferroic heterostructures composed of one magnetic layer and one ferroelectric (multiferroic) layer demonstrated that moderate magnetoelectric coupling can be achieved in such structures.^16−20^ The magnetic part directly breaks the equivalency of the two polarized states of the ferroelectric (multiferroic) materials, leading to enhanced magnetoelectric coupling. However, the modulation by van der Waals (vdW) interaction is too weak to be easily performed,^21−23^ and the fabrication of high-quality 2D vdW heterostructures with enhanced coupling on a large scale is challenging. Therefore, strategies for designing materials with intrinsic inversion symmetry breaking between the two polarized states for robust magnetoelectric coupling are needed. For example, a 2D type I multiferroic double-metal trihalide monolayer, ReWCl_6_, was reported recently.^24^ However, the in-plane polarization reversal in ReWCl_6_ was more difficult than the out-of-plane one, and the Curie temperatures of two phases (C type and D type) were far below room temperature. In addition, an asymmetric multiferroic based on an Fe–Cr–Mo superlattice with the LiNbO_3_-type structure was proposed in 2014,^25^ but it seems to be difficult to apply in other three-dimensional systems.

In this work, Janus 2D multiferroic MXene-analogous oxynitride is designed for realizing multiple and practical magnetoelectric couplings on the basis of first-principles calculations. We find the coexistence of three inequivalent phases (p1–p3) in oxynitride InTlNO_2_, which may lead to feasible multistate memory. p1 and p2 are metallic, and p3 is semiconducting. Importantly, all three phases are multiferroics with *T*_C_ values that are higher than room temperature. There is a 90° rotation of the magnetic anisotropy easy axis between p1 and p2. Therefore, polarization switching can control both the magnetism (*T*_C_) and magnetic anisotropy of InTlNO_2_. Our strategy can be applied to other 2D multiferroics by creating Janus structures.

InTlNO_2_ is achieved by replacing one layer of In (Tl) ions of In_2_NO_2_ (Tl_2_NO_2_) with Tl (In) ions, where X_2_NO_2_ (X = In or Tl) had been reported to be 2D multiferroics with a p-orbital magnetism.^7^ This substitution makes the original polarization states distinct (Figure 1). We denote the polarized structure with nitrogen at the top of Tl as p1, the one with nitrogen at the bottom of In as p2, and the centrosymmetric-like structure as p3. The structures were fully relaxed to obtain lattice constants of 3.468, 3.498, and 3.472 Å for p1–p3, respectively. which are between those of In_2_NO_2_ (3.40 Å) and Tl_2_NO_2_ (3.54 Å). p1 and p2 possess *C*_3*v*_ symmetry, just like their parent compounds. p3 possesses *C*_*s*_ symmetry due to asymmetry-induced distortion. The phonon dispersions and AIMD simulations demonstrate that all three phases are dynamically and thermodynamically stable (Figures S1 and S2). The mechanical stability is also confirmed to be stable because all phases meet the Born–Huang criteria (Table S1).^26^ The coexistence of three inequivalent phases in InTlNO_2_ indicates possible applications of multistate memory.

**Figure 1:**
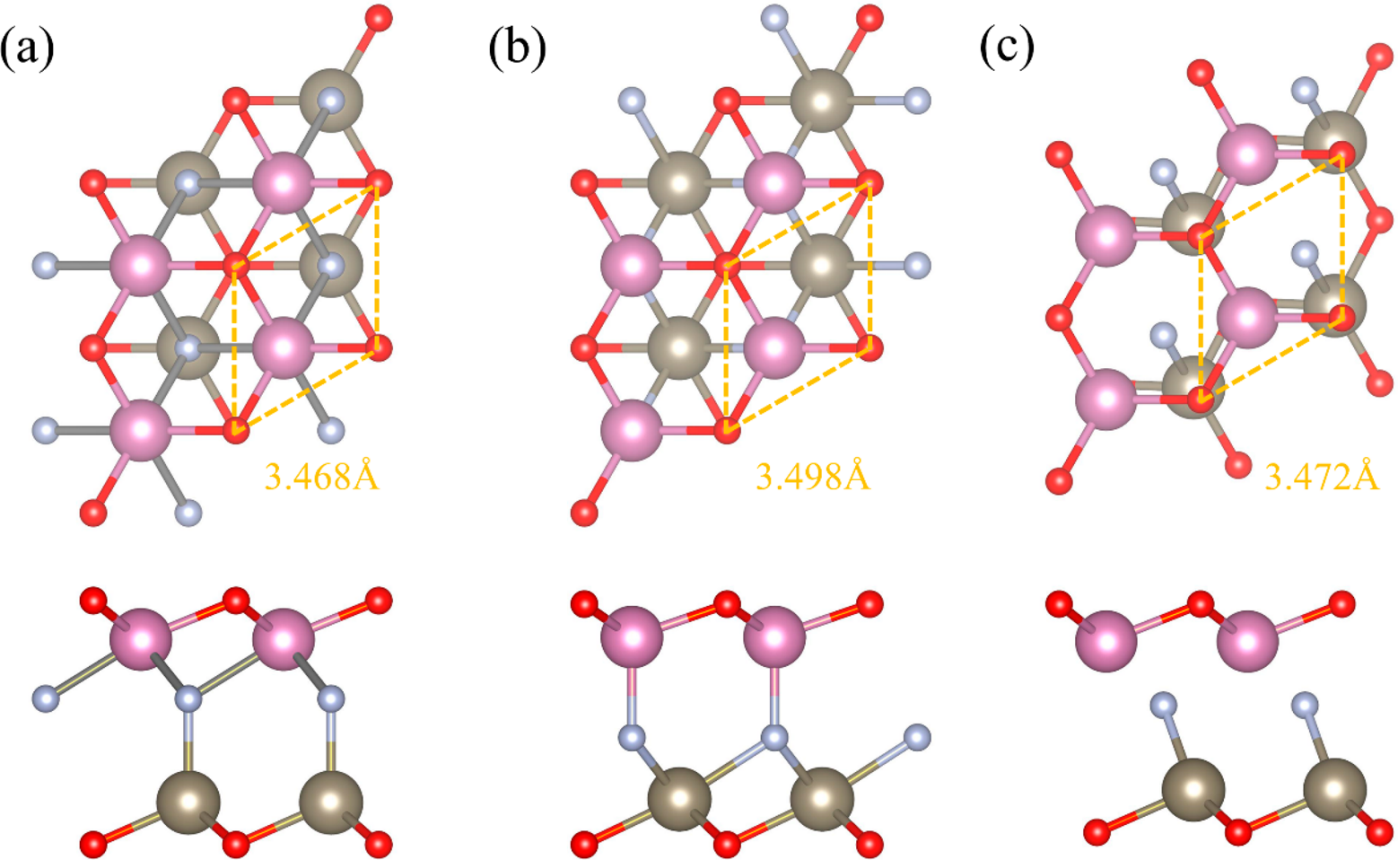
Top and side views of InTlNO_2_ with (a) p1, (b) p2, and (c) p3. Oxygen (red), nitrogen (light gray), indium (pink), and thallium (dark gray).

On the basis of the optimized structures, the magnetic and electronic properties of InTlNO_2_ were investigated. We determined the magnetic ground states for all three phases by considering four different spin configurations (Figure S3), in which the exchange interactions up to the third nearest neighbors were included (details in the Supporting Information).^27,28^ The ferromagnetic states are lowest in energy for all three phases, while the exchange coupling constants behave differently (Table 1). The negative values of *J*_1_ and *J*_2_ account for the ferromagnetic ground states for all three phases. The *J*_2_ of p3 is larger than those of p1 and p2, indicating weak ferromagnetic coupling between the second nearest neighbors of p3. Similar to X_2_NO_2_ (X = In or Tl), all three phases manifest p-orbital magnetism. The central N ion contributes most to the magnetic moment (∼0.6 μ_B_), while the O ion near the N ion also carries a moment of ∼0.2 μ_B_. The total magnetic moments of p1 and p2 are ∼1.01 and ∼1.02 μ_B_ per unit cell, respectively, and that of p3 is exactly 1 μ_B_.

**Table 1:** Nearest-Neighbor (*J*_1_), Second-Nearest-Neighbor (*J*_2_), and Third-Nearest-Neighbor (*J*_3_) Exchange Coupling Constants (millielectronvolts) for p1–p3, Respectively

	*J*_1_	*J*_2_	*J*_3_
p1	–69.5	–44.8	6.5
p2	–53.1	–42.1	8.5
p3	–43.4	–4.5	–13.6

On the basis of the ground states, the band structures of InTlNO_2_ (Figure 2) were studied with the HSE06 hybrid functional. We find that p1 and p2 are half-metallic because their spin-up states are n-type semiconducting as their conduction band minima (CBMs) are lower than the Fermi level at the Γ point by just 0.02 and 0.1 eV for p1 and p2, respectively, while the spin-down bands are metallic.^29−31^ The projected densities of states (PDOSs) (Figure S4) demonstrate the strong coupling between the p_*x*_/p_*y*_ orbitals of N ions and the p_*z*_ orbital of the O ions near the Fermi level, leading to the magnetic moments. For p1 and p2, the ferromagnetic coupling can be explained by the Stoner effect. According to the Stoner model, the itinerant ferromagnetism occurs if *JD*(*E*_F_) > 1 is satisfied, where *D*(*E*_F_) is the DOS at Fermi energy *E*_F_ and *J* denotes the strength of the exchange interaction.^32^*D*(*E*_F_) are 9.52 and 3.78 states/eV for p1 and p2, respectively (Figure R1-3), while *J* is ∼2.5 eV. The value of *J* is slightly larger than those of the previous reports because of the use of the HSE06 functional in our calculations. The half-metallic characteristics also indicate the carrier-mediated double exchange is responsible for the ferromagnetic couplings in both phases.^32−34^ On the contrary, p3 is intrinsically semiconducting as the spin-up and spin-down states have gaps of 1.91 and 0.88 eV, respectively. The PDOSs (Figure S4) show that the states slightly above the Fermi level for both p1 and p2 are mainly composed of the p_*x*_ and p_*y*_ orbitals of the N ions and the p_*z*_ orbitals of the O ions in the spin-down bands, which are relatively flat in the Γ–M path (Figure 2). Interestingly, they shift below the Fermi level and become the valence band maximum (VBM), resulting in the creation of a gap in the spin-down bands of p3. The conduction band minimum of p3 is dominated by the p_*z*_ states of N and O ions (Figure S4). The spin-up band of p3 is quite similar to those of p1 and p2. The p3-InTlNO_2_ structure has two shortened In–N bonds (2.30 Å), two elongated Tl–N bonds (2.98 Å), one elongated In–N bond (2.78 Å), and one shortened Tl–N bond (2.23 Å) compared with those of center-symmetric In_2_NO_2_, leading to the change in the ion charge states as well as the coupling among them, which is responsible for the opened gap in the spin-down channel. Because of the semiconducting nature, the long-range magnetic coupling between the moments in the p3 phase can be attributed to the direct p–p interactions.^35−37^ Moreover, the nearest N–In–N bonding angle is close to 90° (Figure S6), resulting in super-exchange for the ferromagnetic coupling of p3 according to the Goodenough–Kanamori–Anderson (GKA) rules.^38^

**Figure 2:**
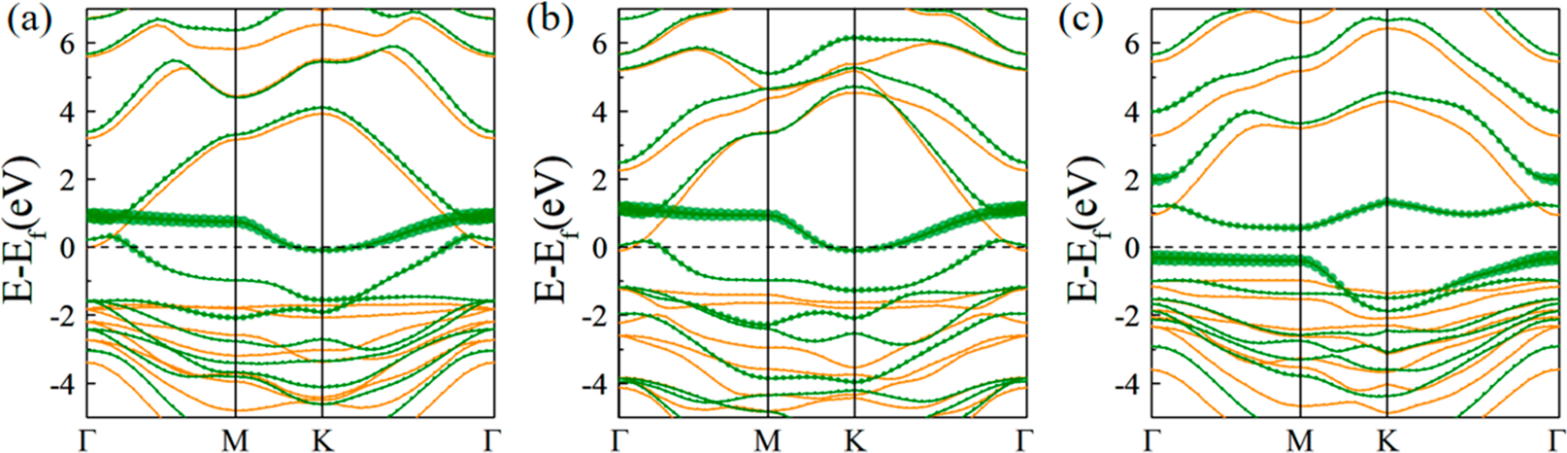
Spin-polarized band structures of (a) p1, (b) p2, and (c) p3 determined with the HSE06 hybrid functional. Orange and olive lines represent the spin-up and spin-down bands, respectively. Projected band structures for the p orbitals of N ions are marked by olive bubbles.

As there are three distinct phases, the phase transition was explored by the nudged-elastic-band method.^39^ Similar to the case for ReWCl_6_,^24^ asymmetric transition paths are expected because all three phases of InTlNO_2_ are inequivalent. The evident imaginary modes in the phonon dispersions of the transition states (Figure S7) indicate their instability. The activation energy barrier from p1 to p2 (p1 → p2) is 108 meV/formula unit (f.u.), while the reverse process (p2 → p1) needs an energy barrier of 53 meV/f.u. Moreover, the transition from p1 to p2 inevitably goes through the intermediate p3 phase. The energy barriers for the p1 → p3, p3 → p1, p3 → p2, and p2 → p3 transitions are 18, 4, 94, and 53 meV/f.u., respectively (Figure 3), which are all comparable with those of previous theoretical reports on ferroelectricity.^7,40−42^ However, the energy barrier between p3 and p1 (4 meV/f.u.) seems to be too small to resist thermal fluctuation at room temperature (26 meV). This concern can be eliminated in an easy way. The InTlNO_2_ monolayer has five distinct sublayers, and the transition between any of the two phases always involves a collective sliding among different sublayers. Therefore, the energy barrier required to achieve the transition should be much larger than our calculated values because more than one primitive cell needs to be considered. Therefore, the energy barrier can be robust enough against thermal fluctuation at room temperature, which is in agreement with our AIMD results (Figure S2).

**Figure 3:**
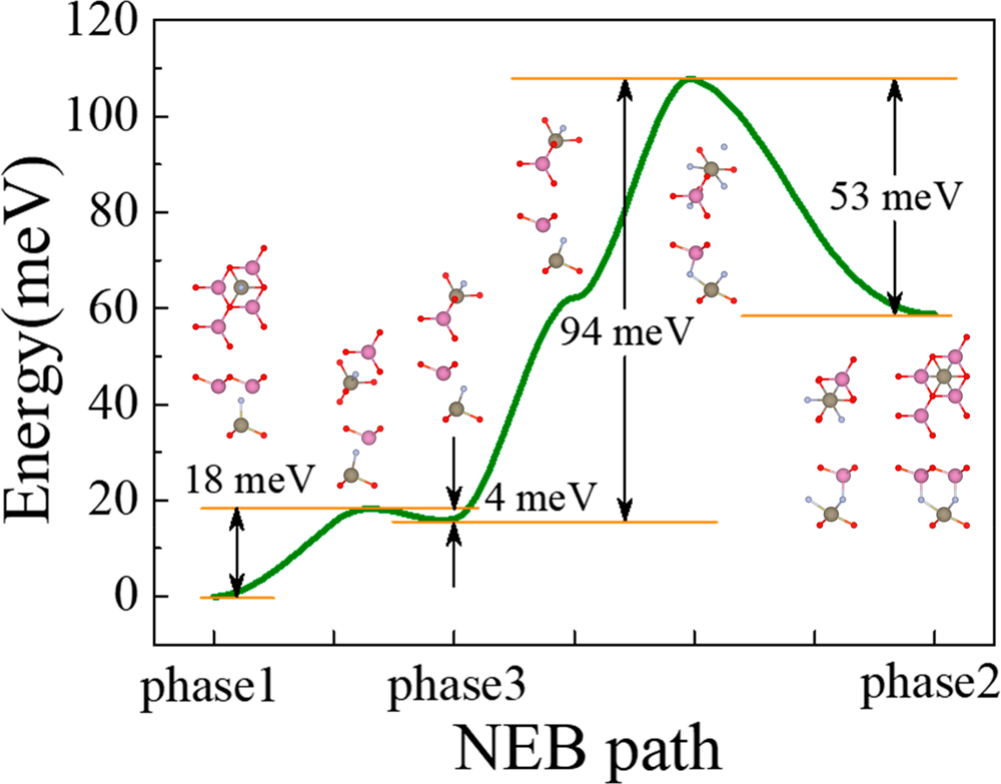
Minimal energy pathway of the transition process for the three different phases.

To determine whether the transformation under an external field is possible, the electric polarization of InTlNO_2_ was investigated on the basis of a reported method.^7,15^ The calculated vacuum level differences (ΔΦ) are 0.26, −2.03, and −0.50 eV, and hence the out-of-plane polarizations are 2.30, −17.97, and −4.43 pC/m, for p1–p3, respectively. Therefore, we can conclude that different phases of InTlNO_2_ exhibit different out-of-plane polarizations, which makes the phase transition among p1–p3 viable by the external electric field. Meanwhile, due to the asymmetric energy barriers, six kinds of transitions need individual consideration (p1 to p2, p2 to p1, p1 to p3, p3 to p1, p2 to p3, and p3 to p2). A detailed discussion of the electric transformation is included in the Supporting Information (Figures S9–S11). We also considered the effect of strain on the relative energy of different phases, which can influence the transformation (Figure S12). When the strain is low, the relative energies among the three phases are not affected. In the orange region, where the strain is more negative than −3% or between 2% and 5%, p3 becomes the most stable phase. When the tensile strain is >5%, the relative energy between p1 and p2 was reversed, while p3 is still the most stable phase. Therefore, we conclude that the strain may have a pronounced effect on the transition of different phases because of its evident influence on the relative energy of p1–p3.

To determine the Curie temperature, we carried out the MC simulations based on the classical Heisenberg model.^27^ The specific heat curves (Figure 4a) indicate that the Curie temperatures of p1–p3 are around 420, 336, and 274 K, respectively. Additionally, our AIMD results (Figure S2) manifest that the magnetism is well preserved at 300 K for all three phases, suggesting some underestimation for the Curie temperatures from our MC simulations, but the results of MC simulations are qualitatively correct considering the ferromagnetic coupling of p1 is strongest while that of p3 is weakest from the calculated exchange parameters presented above. Therefore, we can tune the Curie temperature through phase transition by introducing an external electric field. For example, the working temperature range can be between the Curie temperatures (from 336 to 420 K) of p1 and p2 for the p1 → p2 transition, where the magnetism of p1 is always stronger than that of p2 (Figure 4b). Therefore, the electrically induced transition between p1 and p2 can always change the magnetic state.

**Figure 4:**
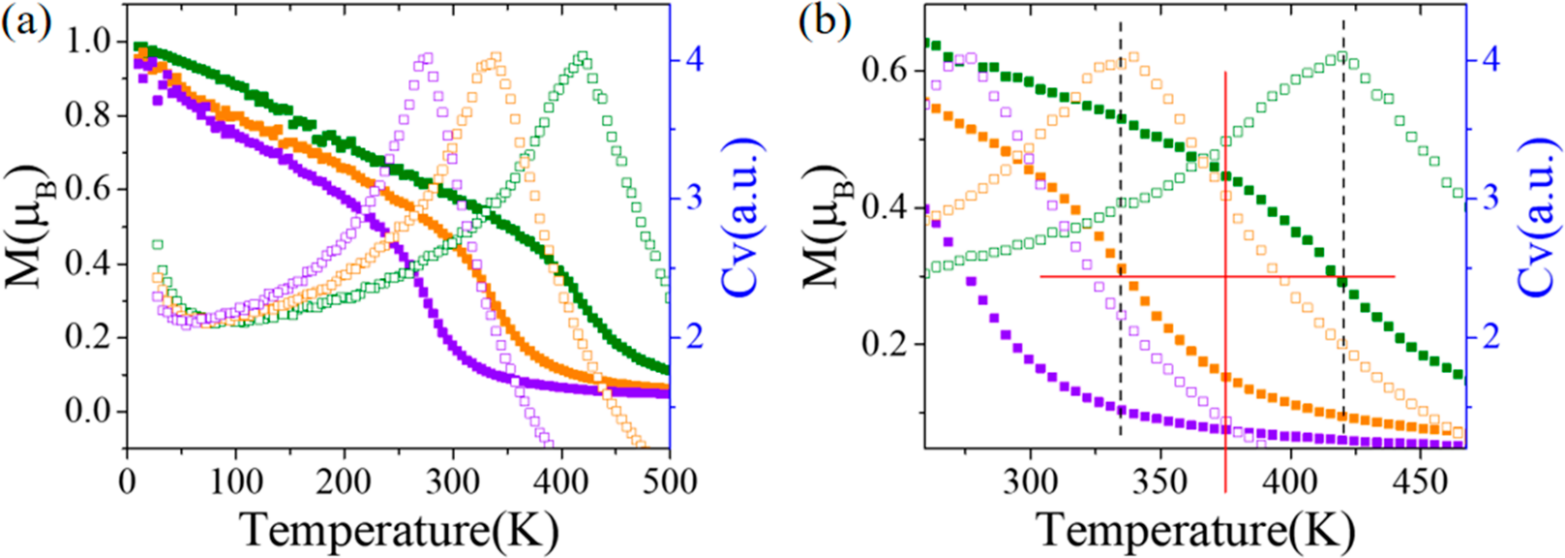
(a) Temperature dependence of magnetic moment *M* (filled squares) and specific heat *C_v_* (empty squares) of p1 (olive), p2 (orange), and p3 (violet). (b) Enlarged images of panel a showing the origins of the operating temperature of the potential devices.

In addition, the controlled magnetic anisotropy during the phase transition may make InTlNO_2_ suitable for practical application at room temperature. By calculating the angle-dependent magnetic anisotropic energies with spin–orbital coupling (SOC) in the *x–z*, *y–z*, and *x–y* planes, we demonstrate that the modulation of in-plane to out-of-plane magnetic anisotropy can be easily achieved. We define the magnetic anisotropy energy (MAE) as the difference in energy per N ion between the easy-axis and hard-axis directions. The radius of each point (Figure 5) represents the absolute value of energy when the magnetic moment is fixed in the corresponding direction (θ). The larger the radius, the lower the energy. We see that p1 exhibits an easy magnetic *x–y* plane with an MAE of 87 μeV, while the easy axis of p2 favors the out-of-plane direction with an MAE of 135 μeV, values that are comparable to those of reported ferromagnetic 2D materials.^43^ Therefore, a controllable magnetic anisotropy is expected through the transition between p1 and p2. We find that p1 exhibits electronic and magnetic properties similar to those of Tl_2_NO_2_, while p2 and In_2_NO_2_ have an out-of-plane easy axis. The magnetic anisotropies of p1 and p2 are attributed to the d orbitals of In and Tl. The orbital-resolved Δ*E*_soc_ can be used to identify the contribution, where positive and negative values represent out-of-plane and in-plane magnetic anisotropy, respectively.^44^ The interactions between the d orbitals of In and Tl ions contribute most to the total magnetic anisotropy (Figures S13 and S14), while those of p orbitals and other ions are negligible. For p1, the interactions between the d_*xz*_ and d_*yz*_ orbitals of Tl ions and the d_*xy*_ and d_*x*^2^–*y*^2^_ orbitals of In ions contribute to the in-plane magnetic anisotropy. For p2, the magnetic anisotropy is dominated by the interaction among the d orbitals of the Tl ions. The interactions between the d_*yz*_ and d_*z*^2^_ (d_*xy*_ and d_*x*^2^–*y*^2^_) are stronger than that between the d_*xz*_ and d_*yz*_ orbitals, rendering p2 with out-of-plane magnetic anisotropy. p3 shows relatively weak magnetic anisotropy with an MAE of 40 μeV (Figure S15), whose easy axis lies on the *y–z* plane with an angle of 116° to the *z* axis. We find that p3 favors the in-plane direction due to the interactions between the p orbitals of both Tl and In ions (Figures S16 and S17), while the deviated easy axis from the in-plane direction originates from the interactions between the p_*x*_ and p_*z*_ orbitals of the In ions (Figures S18 and S19).

**Figure 5:**
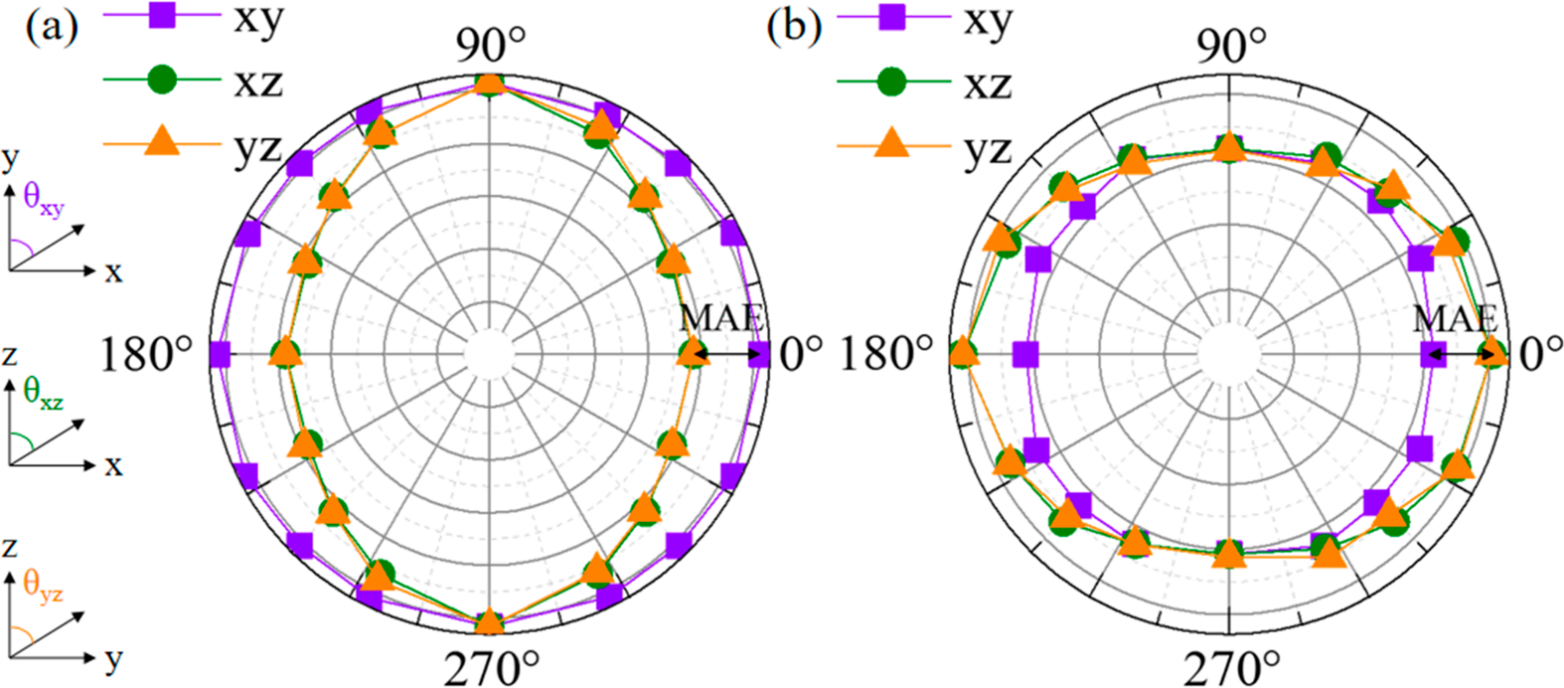
Angle-dependent magnetic anisotropic energies of (a) p1 and (b) p2 with magnetic moments lying on the *x–y* (violet), *x–z* (olive), and *y–z* (orange) planes. The definition of angles is shown in the left. The MAE is marked by the arrows.

Although InTlNO_2_ is similar to the parent structure X_2_NO_2_ (X = In or Tl), the X_2_NO_2_ (X = In or Tl) compounds provide the p-orbital multiferroics for the incompatibility between the d-orbital magnetism and d^0^ ferroelectricity in traditional multiferroics, while it is InTlNO_2_ for enhanced magnetoelectric coupling. Our strategy may pave the way for the incompatibility between prominent magnetoelectric coupling and considerable polarization.

To make our design more reasonable, we compare the energies of three different phases (Figure S20) that may compete with the Janus InTlNO_2_ during the fabrication and find that their energies are relatively similar (Table S2). Therefore, a three-step method is proposed that may accurately synthesize the Janus InTlNO_2_ on the basis of previous successful examples.^45,46^ The first step is the fabrication of In_2_N (Tl_2_N). Given the successful synthesis of two-dimensional titanium nitride Ti_4_N_3_ (MXene)^46^ since 2016, the fabrication of In_2_N (Tl_2_N) can use a similar molten salt treatment. The second step is the one-layer substitution that transforms In_2_N (Tl_2_N) into InTlN, which may be achieved by either hydrogen plasma-assisted substitution^47^ or fine control of the temperature during the substitution process.^48^ The last step is to terminate the obtained pure InTlN with functional groups, which are oxygen atoms here. The functionalization should naturally occur under the ambient condition, after which the Janus InTlNO_2_ can be obtained.

In summary, Janus 2D asymmetric multiferroics are demonstrated to achieve multiple and practical magnetoelectric coupling on the basis of first-principles calculations. We find that 2D Janus InTlNO_2_ has three inequivalent phases with variable electronic and magnetic properties, which pave the way for multiple-state memory devices. We show that p1-InTlNO_2_ and p2-InTlNO_2_ are half-metallic while p3-InTlNO_2_ is a semiconductor. All three phases exhibit room-temperature ferromagnetism with the Curie temperature decreasing from p1 to p2 to p3. We further show that the magnetic anisotropy can be controlled by an external electric field. Our findings may provide a novel strategy for the design of 2D multiferroics for practical applications.

## Methods

Our density functional theory (DFT) calculations^49^ were performed using the Vienna Ab Initio Simulation Package (VASP) codes.^50^ The electron–ion interaction was treated by the projector-augmented wave method.^51^ The Perdew–Burke–Ernzerhof (PBE) version of the generalized gradient approximation (GGA) was used for the exchange-correlation functional.^52^ The Heyd–Scuseria–Ernzerhof hybrid functional (HSE06) was employed to obtain accurate electronic band structures.^53^ A vacuum region of >15 Å along the *z* direction was set to avoid the interaction between the neighboring layers. The plane-wave cutoff energy was set as 520 eV. The 16 × 16 × 1 and 12 × 12 × 1 Γ-centered Monkhorst–Pack k-meshes were used for the PBE and HSE06 calculations, respectively.^54^ The convergence criteria for energy and ionic relaxation were 10^–8^ eV and 10^–3^ eV/Å, respectively. The phonon dispersions were calculated on the basis of the density functional perturbation theory (DFPT) method^55^ using the PHONONPY code.^56^ Ab initio molecular dynamics (AIMD) simulations were carried out with a 5 × 5 × 1 supercell at 300 K with a time step of 1 fs. The climbing image nudged-elastic-band (CI-NEB) method^39^ was implemented to calculate the energy barriers during the phase transitions. The Monte Carlo (MC) simulations based on the Heisenberg model were performed using MCSOLVER to estimate the Curie temperature.^28^ A 2D 50 × 50 × 1 hexagonal spin–lattice accommodating 2500 local magnetic moments with periodic boundary conditions was used in our Monte Carlo simulations. During the simulations, each spin could rotate randomly and isotropically. Such a process lasted for 3 × 10^7^ loops at each temperature. The average magnetization per site (*M*) and the specific heat, defined as , were sampled after 1 × 10^7^ loops, which was large enough to ensure a good thermodynamic equilibrium. VASPKIT was included for data postprocessing.^57^

## Supporting Information Available

Additional information, including Figures S1–S20 and Tables S1 and S2, and additional references (PDF)
Transparent Peer Review report available (PDF)
