# Supplementary material for: Two-Dimensional Asymmetric Multiferroics: Unique Way toward Strong Magnetoelectric Coupling and Multistate Memory
Source: J Phys Chem Lett. 2024 Feb 8;15(7):1795–801. doi: 10.1021/acs.jpclett.3c03527 (PMC10895667; doi:10.1021/acs.jpclett.3c03527)
Supplement: Supplementary file 1 — jz3c03527_si_001.pdf [file jz3c03527_si_001.pdf]

# Two-dimensional Asymmetric Multiferroics: Unique Way Toward Strong Magnetoelectric Coupling and Multi-State Memory

Zhichao Yu[1], Haoyun Bai[1], Bowen Li[2], Lun Li[1], Hui Pan[1,3*]

[1] Institute of Applied Physics and Materials Engineering, University of Macau, Macao SAR 999708, P. R. China

[2] 2027 Laboratory, Tianfu Xinglong Lake Laboratory, Chengdu, Sichuan, 610000, P. R. China

[3] Department of Physics and Chemistry, Faculty of Science and Technology, University of Macau, Macao SAR 999078, P. R. China

*Corresponding Author:

H. Pan: huipan@um.edu.mo (email), +853 88224427 (tel.), +853-88222454 (fax)

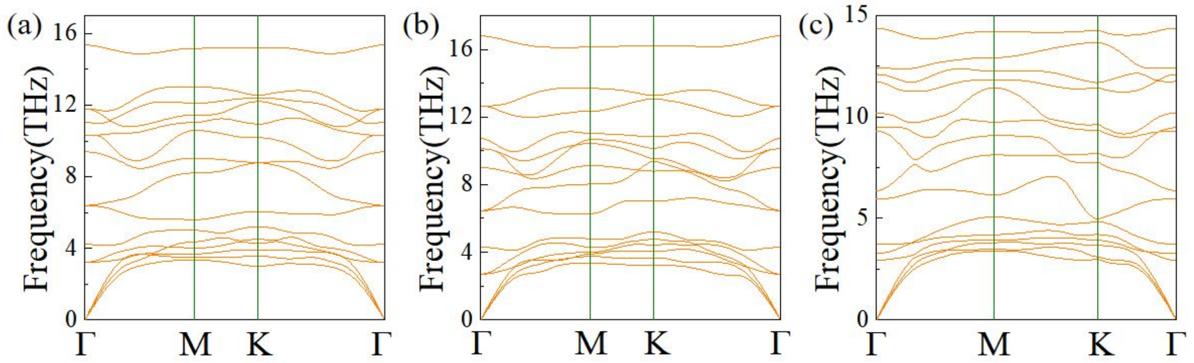


**Figure S1.** The phonon spectra of $InTlNO_2$ monolayer with (a) phase1, (b) phase2 and (c) phase3.

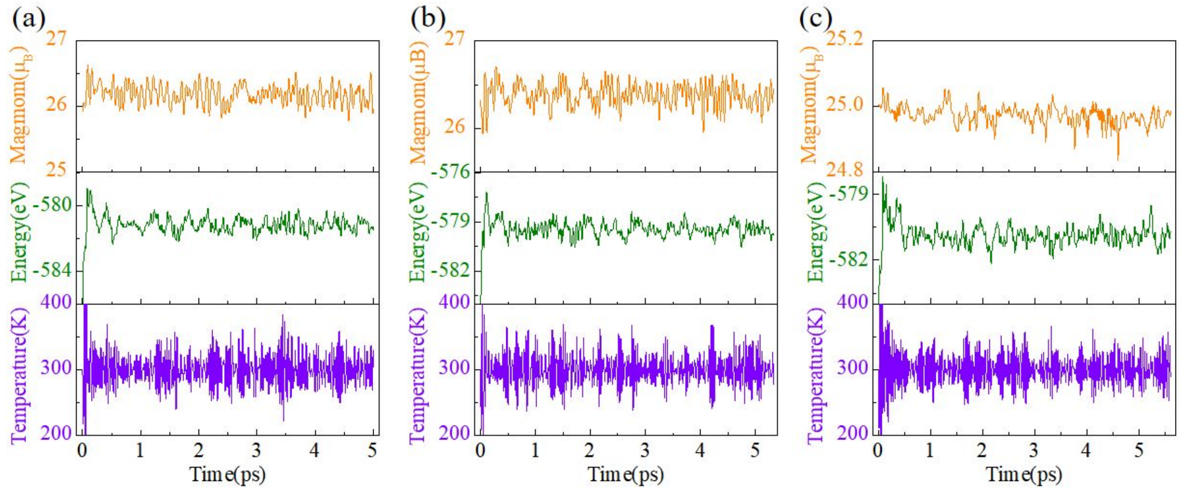


**Figure S2.** AIMD results of (a) phase1, (b) phase 2 and (c) phase 3. The evolution of magnetic moment (orange), total energy (olive) and temperature (violet) against time.

**Table S1.** In-plane elastic constants of phase1, phase2 and phase 3 (kBar).

| Structure | $C_{11}$ | $C_{12}$ | $C_{22}$ | $C_{66}$ |
|---|---|---|---|---|
| phase1 | 460 | 191 | 460 | 134 |
| phase2 | 460 | 185 | 460 | 137 |
| phase3 | 345 | 116 | 325 | 99 |

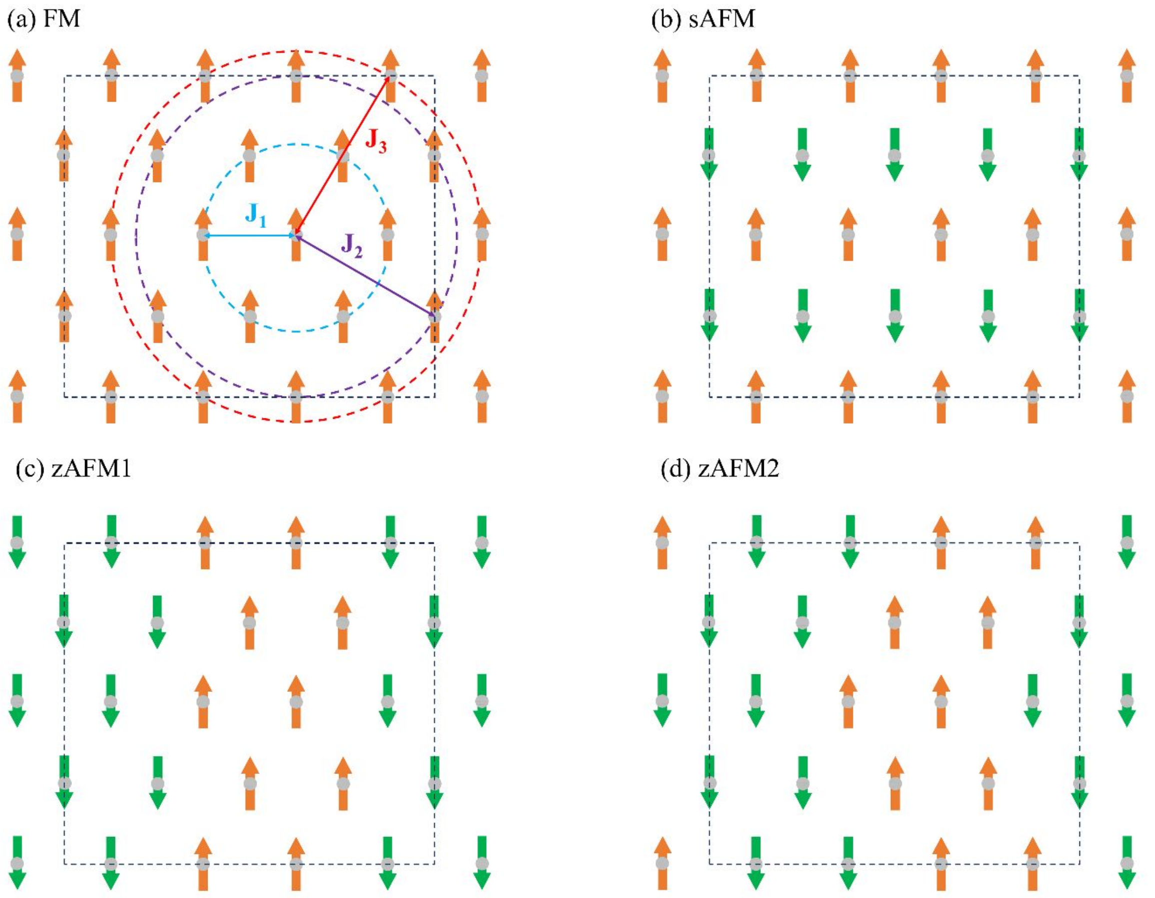


**Figure S3.** The sketch maps of four different spin configurations. (a) FM, (b) sAFM, (c) zAFM1 and (d) zAFM2, respectively.

Considering the nearest-neighbor, second-nearest-neighbor and third-nearest-neighbor magnetic exchange interaction, the spin Hamiltonian can be defined as[1-3] :

$$H = \sum_{m}\sum_{n \in N_n} J_1 \boldsymbol{S_m} \cdot \boldsymbol{S_n} + \sum_{P}\sum_{q \in N'_p} J_2 \boldsymbol{S_p} \cdot \boldsymbol{S_q} + \sum_{k}\sum_{l \in N'_k} J_3 \boldsymbol{S_k} \cdot \boldsymbol{S_l} + \sum_{i} A(S_i^z)^2$$

Where $\boldsymbol{S_m}$ is the spin operator of the site $m$ with $|\boldsymbol{S_m}| = S$, and $S_m^z$ represents the z component of $\boldsymbol{S_m}$. The summation runs over all magnetic sites of a given lattice. $N_n$, $N'_p$ and $N''_k$ are the set of the nearest-neighbor, second-nearest-neighbor and third-nearest-neighbor sites around site m, respectively. $J_1$, $J_2$ and $J_3$ denote the nearest-neighbor, second-nearest-neighbor and third-nearest-neighbor exchange coupling constants, respectively. $A$ is the single-site magnetic anisotropy energy parameter. The total energies of these four spin configurations can be described as:

$$E_{FM} = E_0 + 8S^2\,(6J_1 + 6J_2 + 6J_3)$$
$$E_{sAFM} = E_0 + 8S^2\,(-2J_1 - 2J_2 + 6J_3)$$
$$E_{zAFM1} = E_0 + 8S^2\,(2J_1 - 2J_3)$$
$$E_{zAFM2} = E_0 + 8S^2\,(2J_1 - J_2 - 2J_3)$$

Where $E_0$ represents the energy without magnetic coupling. $E_{FM}$, $E_{sAFM}$, $E_{zAFM1}$ and

$E_{zAFM2}$ are the energies of FM. sAFM, zAFM1 and zAFM2 spin configurations. Therefore, one can obtain:

$$J_1 = -\frac{E_{FM} - E_{sAFM} - 8E_{zAFM1} + 8E_{zAFM2}}{64S^2}$$

$$J_2 = \frac{E_{zAFM1} - E_{zAFM2}}{8S^2}$$

$$J_3 = \frac{E_{FM} + E_{sAFM} - 6E_{zAFM1} + 4E_{zAFM2}}{128S^2}$$

Assuming that S = 1/2 for all, we can obtain the values of these exchange coupling constants.

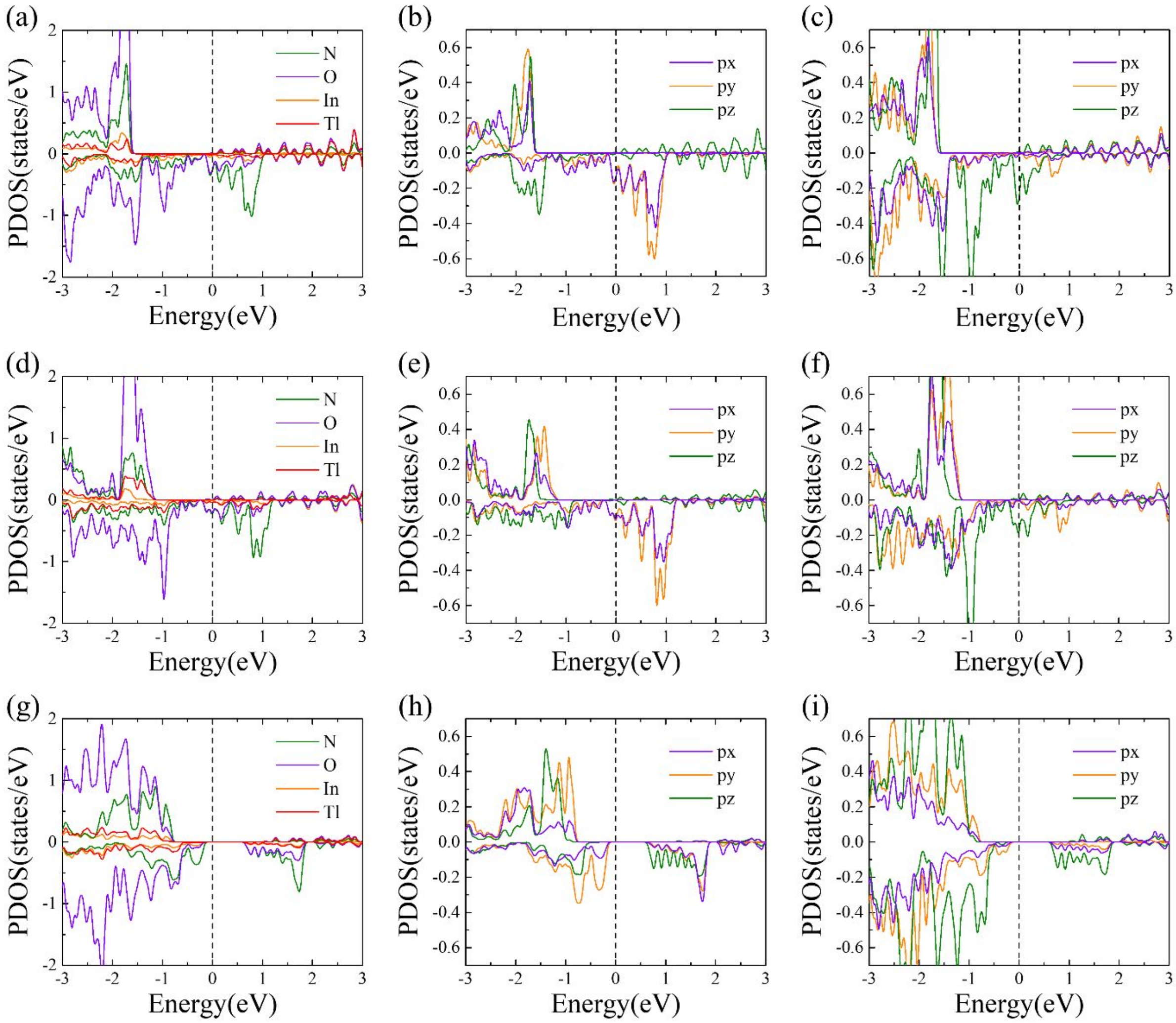


**Figure S4.** Projected density of states (PDOS) of $InTlNO_2$. PDOS for each element of (a) p1, (d) p2 and (g) p3; PDOS for N anions' p orbitals of (b) p1, (e) p2 and (h) p3; PDOS for O anions' p orbitals of (c) p1, (f) p2 and (i) p3.

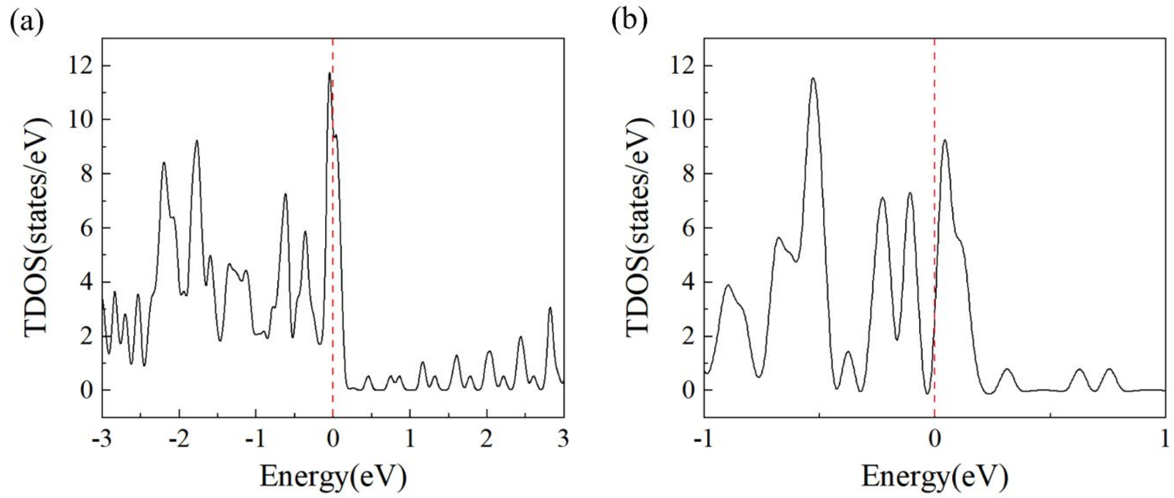


**Figure S5.** Non-spin polarized TDOS of (a) p1 and (b) p2.

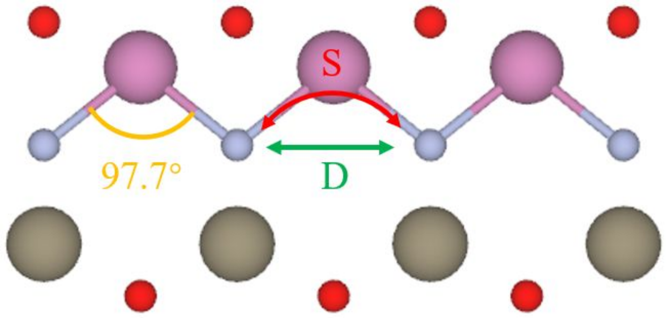


**Figure S6.** A schematic diagram of magnetic exchange interaction in p3 (D: direct exchange, S: super exchange).

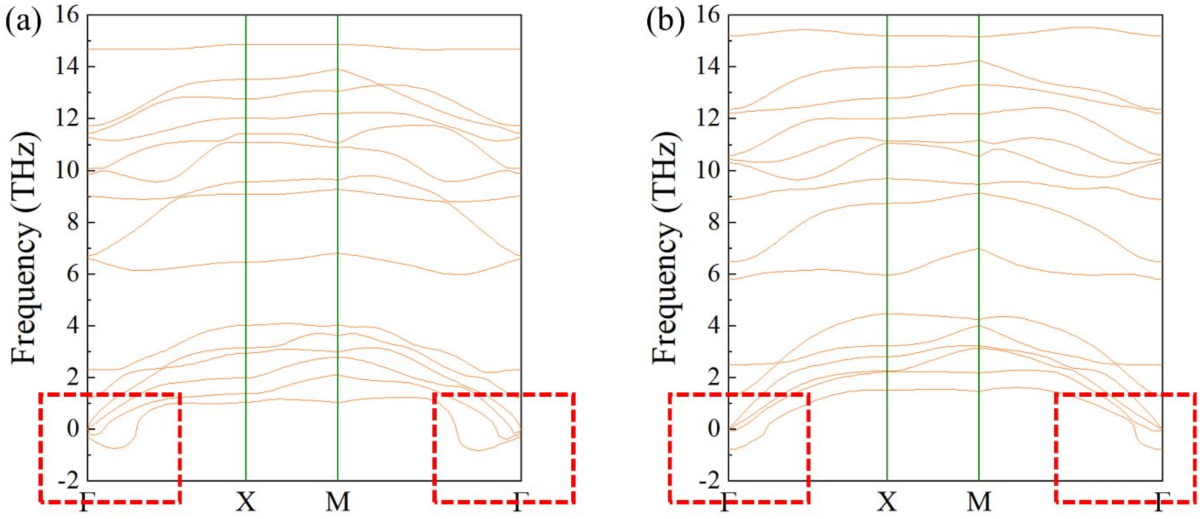


**Figure S7.** The phonon spectra of (a) p12 and (b) p23 (The imaginary modes are marked by red dashed box).

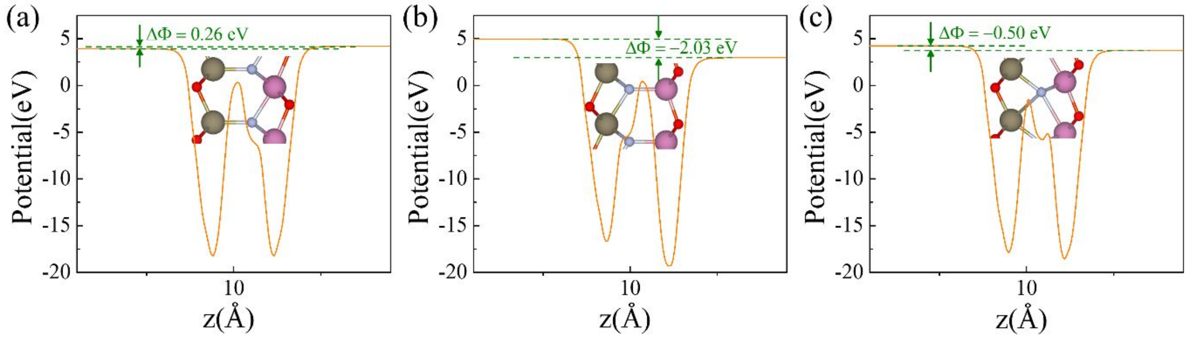


**Figure S8.** Electrostatic potential of p1, p2 and p3.

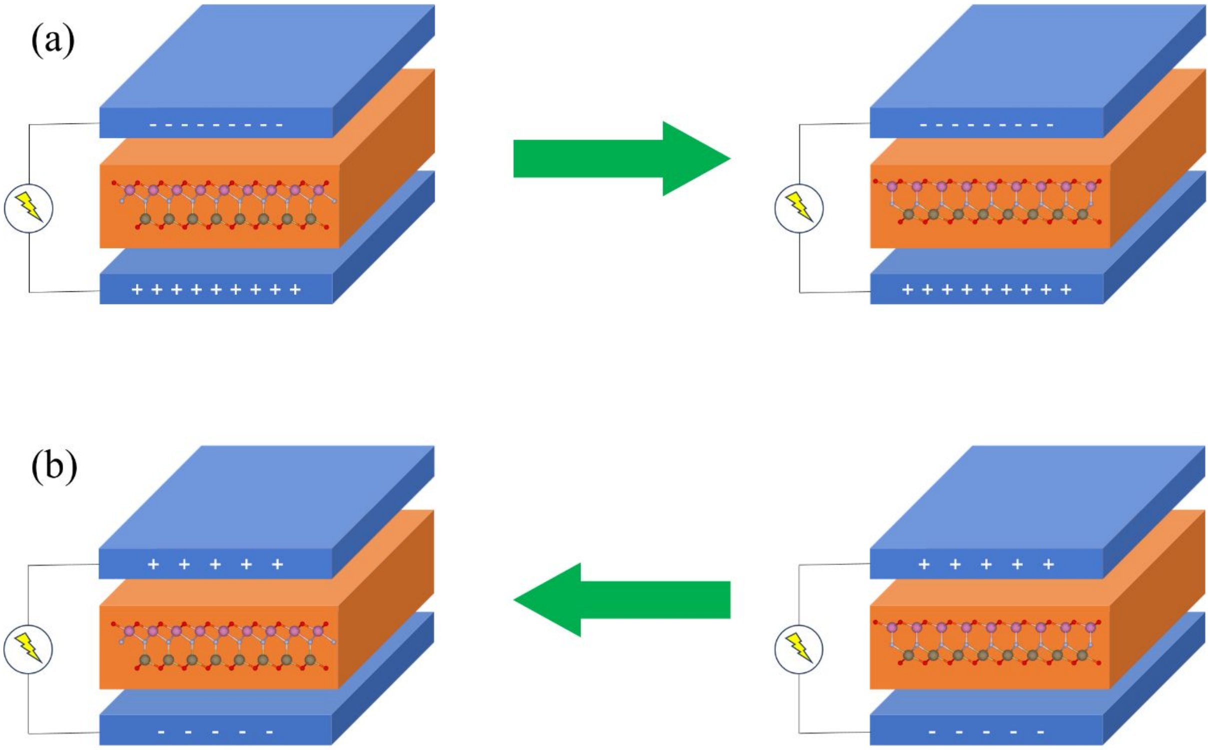


**Figure S9.** (a) p1 to p2 and (b) p2 to p1 (The number of symbols is directly proportional to the electric field strength same as below).

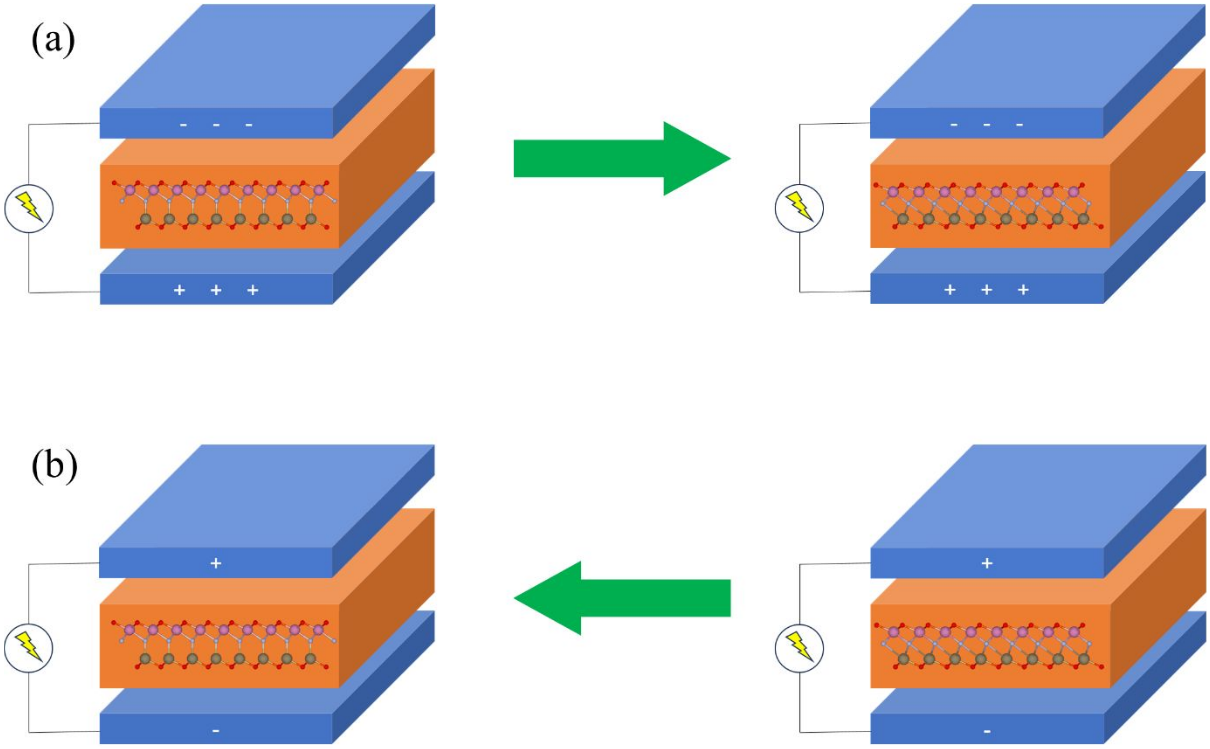


**Figure S10.** (a) p1 to p3 and (b) p3 to p1.

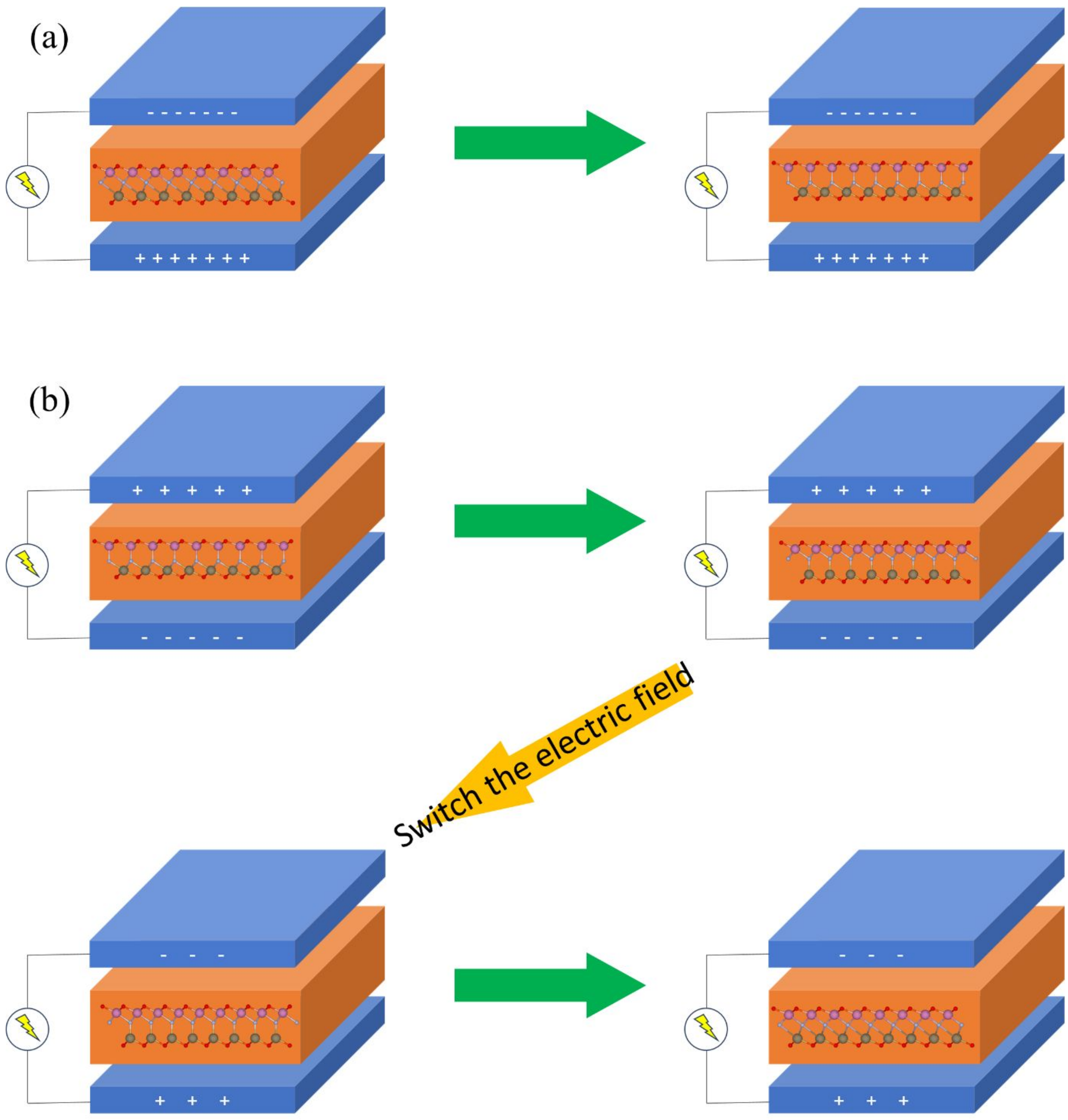


**Figure S11.** (a) p3 to p2 and (b) p2 to p3 (p2→p1→p3).

Due to the asymmetric energy barriers, six kinds of transitions were considered (p1 to p2, p2 to p1, p1 to p3, p3 to p1, p2 to p3 and p3 to p2). According to the CI-NEB curve and the calculated electric polarization, p1 can switch to p2 directly under a large positive electric field, while a medium negative electric field is required to transform p2 back to p1 (**Figure S9**). For p1 and p3, small external field can achieve the transformation between them. p1 can directly cross p3 and transform into p2 if the electric field is large enough to overcome the energy barrier between p3 and p2 (**Figure S10**). The scenario for p2 and p3 may be a little more complicated (**Figure S11**). Relatively large positive electric field is demanded to directly transform p3 to p2, while a negative electric field may always transform p2 to p1 first since the energy barrier for p3 to p1 is much smaller. Therefore, if we want to transform p2 into p3, a negative electric field is required first to switch p2 to p1, and then a smaller positive electric field can transform p1 to p3 (p2→p1→p3).

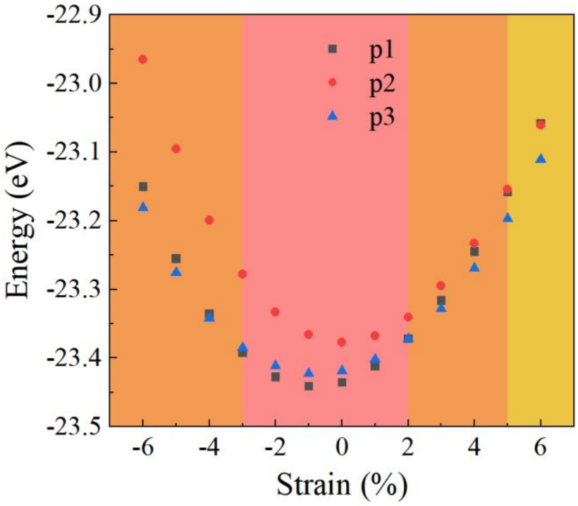


**Figure S12.** Relative energy of p1, p2 and p3 under strain (-6% to 6%).

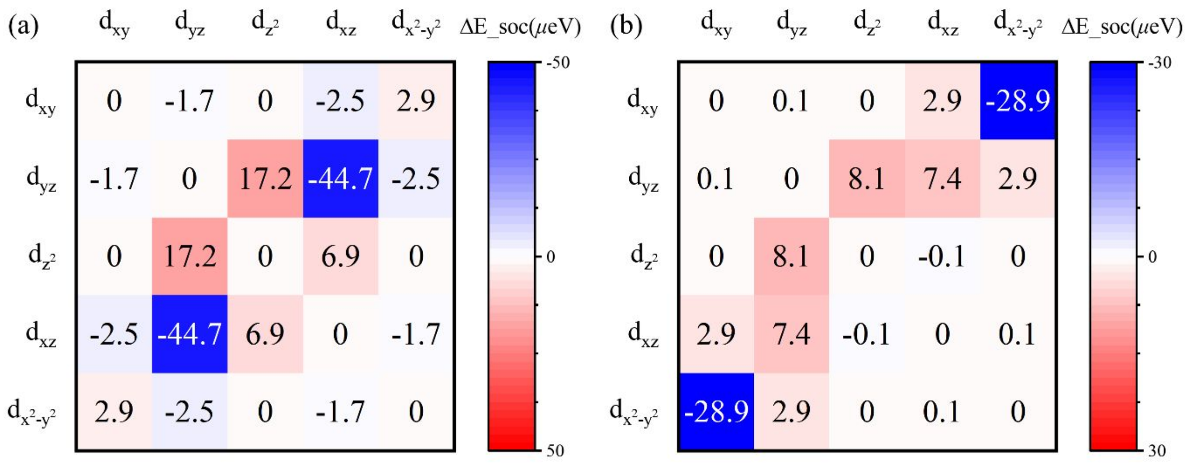


**Figure S13.** Orbital-resolved $\Delta E_{soc}$ of d orbitals for p1: (a) Tl and (b) In.

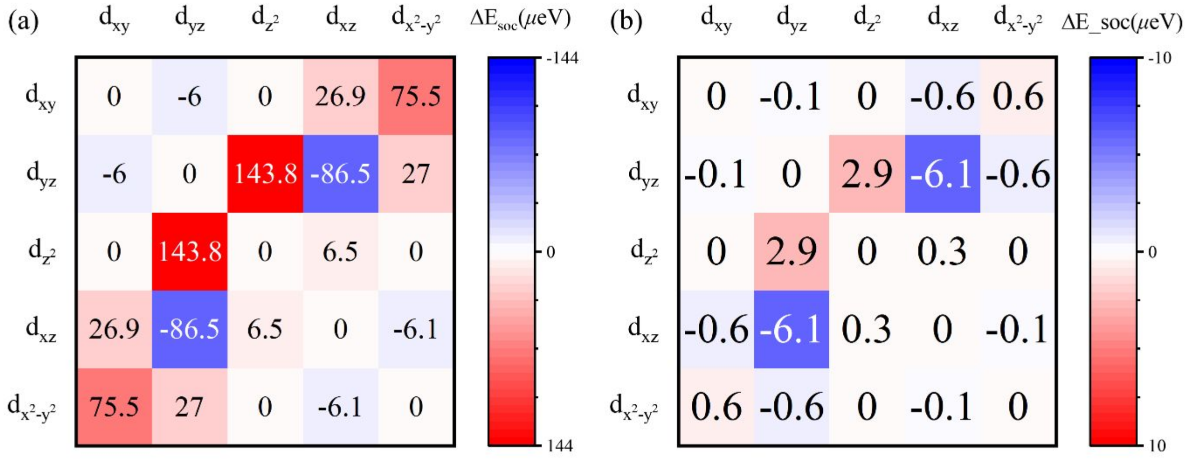


**Figure S14.** Orbital-resolved $\Delta E_{soc}$ of d orbitals for p2: (a) Tl and (b) In.

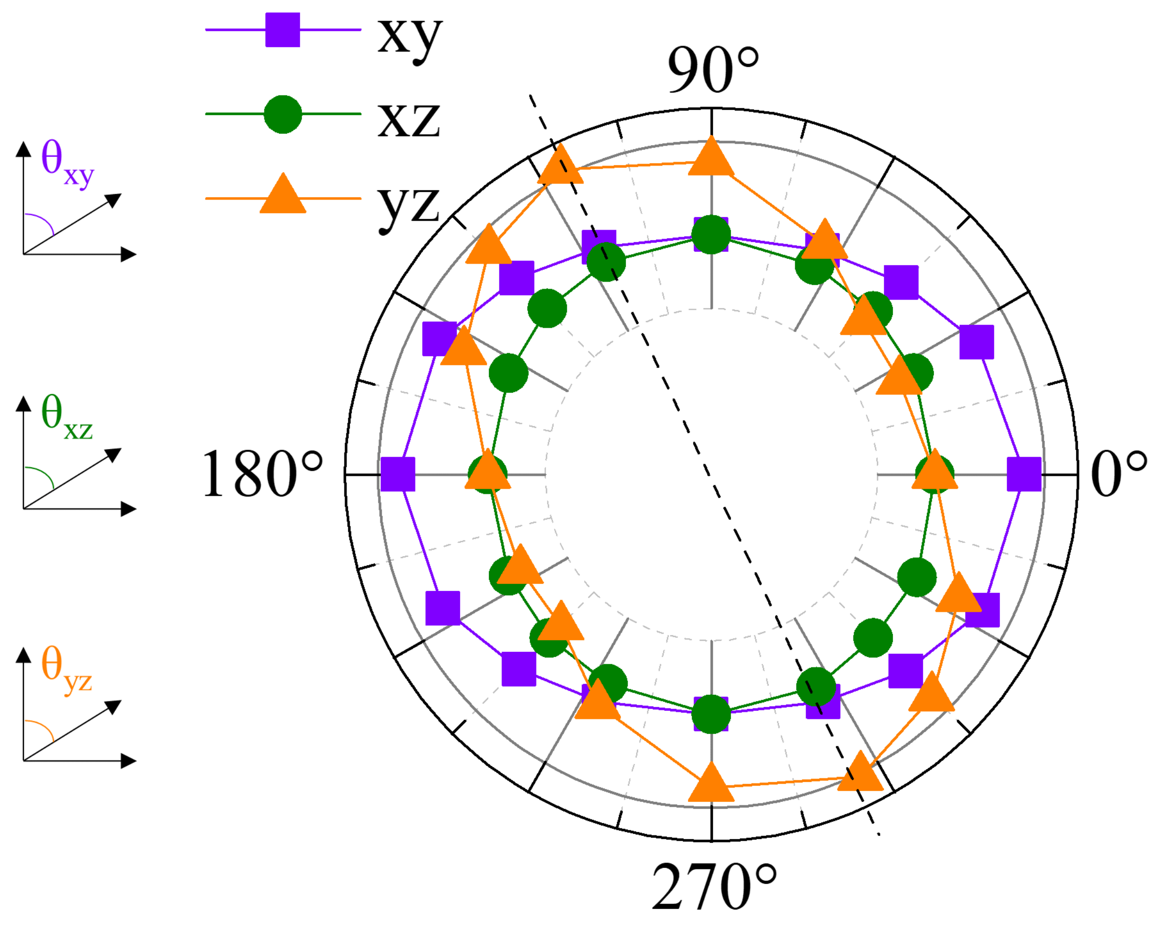


**Figure S15.** Angle-dependent magnetic anisotropic energy of p3 with magnetic moments lying on xy (violet), xz (olive) and yz (orange) planes. The definition of angles is shown in the left.

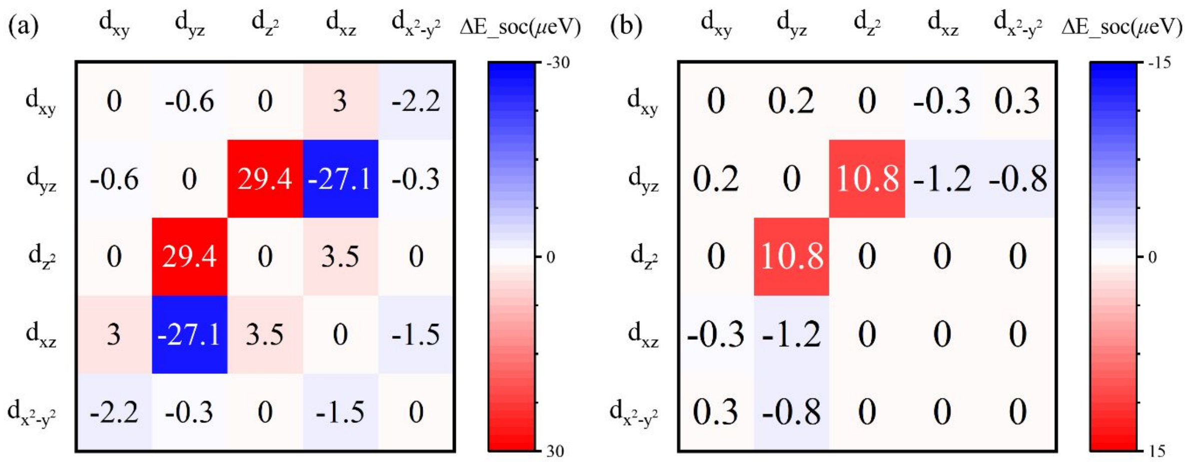


**Figure S16.** Orbital-resolved $\Delta E_{soc}$ of d orbitals for p3: (a) Tl and (b) In.

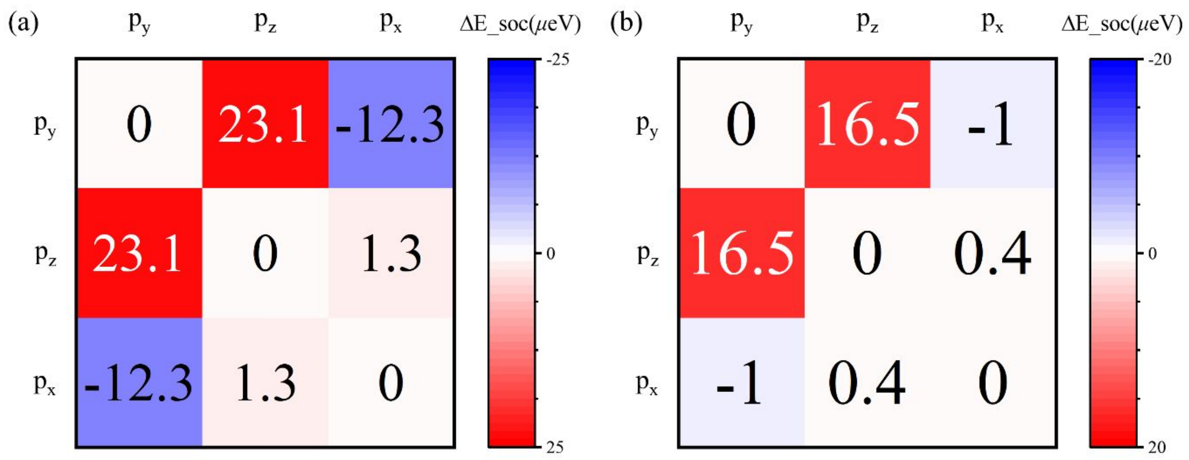


**Figure S17.** Orbital-resolved $\Delta E_{soc}$ of p orbitals for p3: (a) Tl and (b) In.

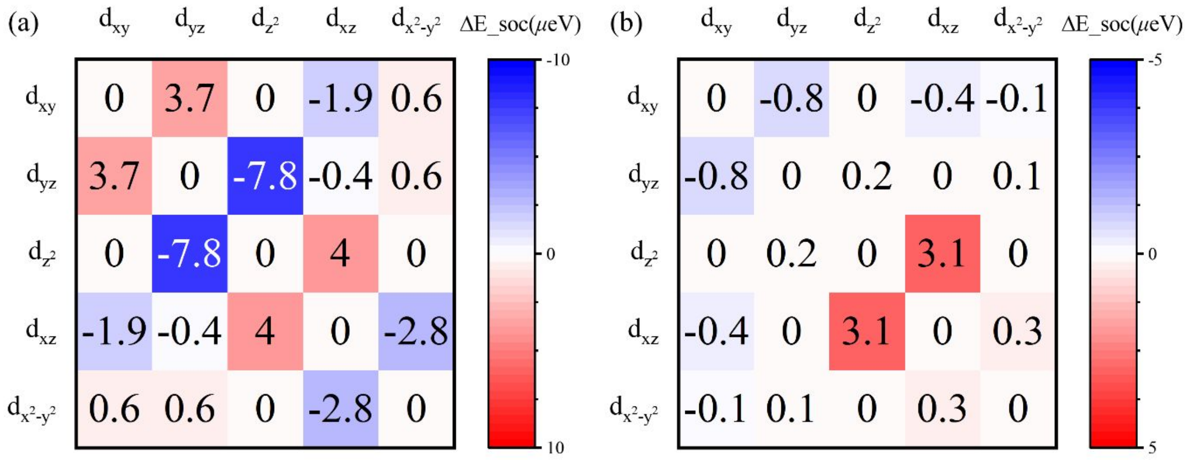


**Figure S18.** Orbital-resolved $\Delta E_{soc}$ (deviation between in-plane direction and easy axis) of d orbitals for p3: (a) Tl and (b) In (Positive values favor the easy axis, while negative values favor in-plane direction).

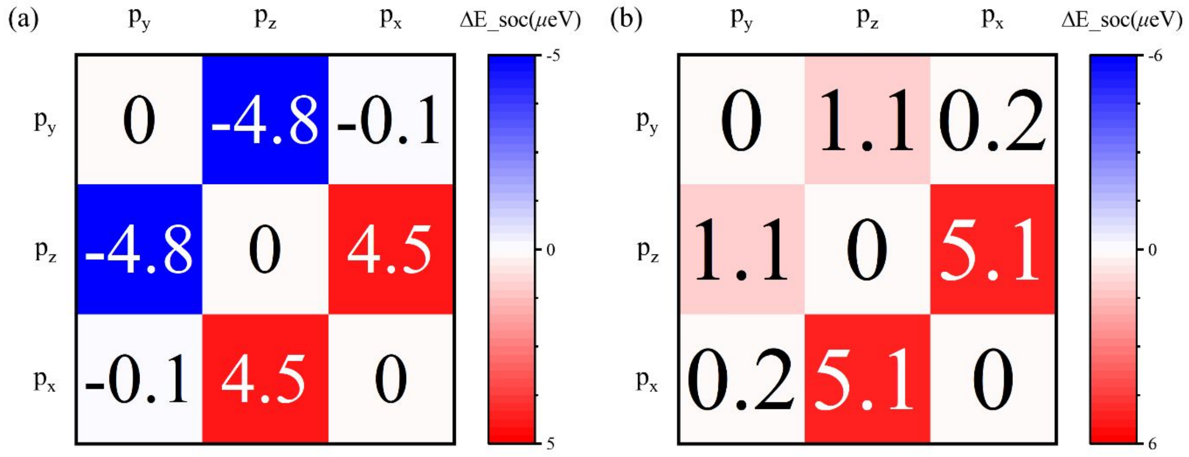


**Figure S19.** Orbital-resolved $\Delta E_{soc}$ (deviation between in-plane direction and easy axis) of p orbitals for p3: (a) Tl and (b) In (Positive values favor the easy axis, while negative values

favor in-plane direction).

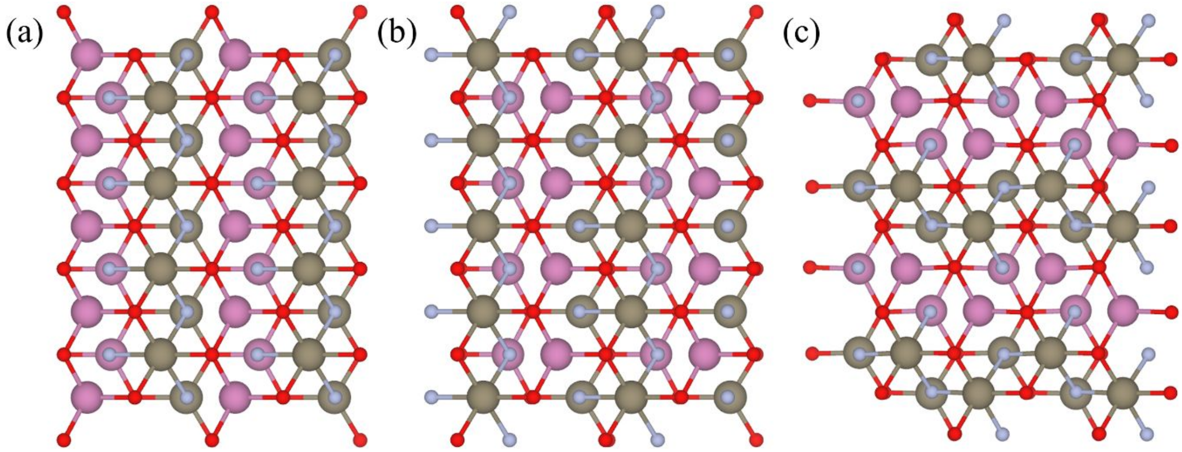


**Figure S20.** Three potential competing phases of $InTlNO_2$: (a) zigzag, (b) parallel and (c) armchair.

**Table S2.** Relative energy of different $InTlNO_2$ (the energy of p1 is set to 0).

| Phase | p1 | p2 | p3 | zigzag | parallel | armchair |
|---|---|---|---|---|---|---|
| Relative energy (meV/atom) | 0 | 12 | 4 | -1 | -2 | -3 |

References


1. Ai, H.; Li, F.; Bai, H.; Liu, D.; Lo, K. H.; Yang, S. A.; Kawazoe, Y.; Pan, H., Ferroelectricity coexisted with p-orbital ferromagnetism and metallicity in two-dimensional metal oxynitrides. *npj Computational Materials* **2022,** *8* (1).

2. Deng, J.; Guo, J.; Chen, X., Molecular Oxygen-Induced Ferromagnetism and Half-Metallicity in alpha-BaNaO4: A First-Principles Study. *J. Am. Chem. Soc.* **2020,** *142* (11), 5234-5240.

3. Liu, L.; Chen, S.; Lin, Z.; Zhang, X., A Symmetry-Breaking Phase in Two-Dimensional FeTe(2) with Ferromagnetism above Room Temperature. *J Phys Chem Lett* **2020,** *11* (18), 7893-7900.
