# Supplementary material for: Two-Dimensional Asymmetric Multiferroics: Unique Way toward Strong Magnetoelectric Coupling and Multistate Memory
Source: J Phys Chem Lett. 2024 Feb 8;15(7):1795–801. doi: 10.1021/acs.jpclett.3c03527 (PMC10895667; doi:10.1021/acs.jpclett.3c03527)
Supplement: Supplementary file 2 — jz3c03527_si_002.pdf [file jz3c03527_si_002.pdf]

jz-2023-03527c.R1

Name: Peer Review Information for "Two-dimensional Asymmetric Multiferroics: Unique Way Toward Strong Magnetoelectric Coupling and Multi-State Memory"

First Round of Reviewer Comments

Reviewer: 1

Comments to the Author

Yu et al. proposed a novel Janus 2D multiferroic MXene-analogous oxynitrides ($InTlNO_2$) based on the first-principles calculations. This Janus structure is different from the previous Janus monolayers, such as MoSSe. Three inequivalent phases can be found, exhibiting potential magnetoelectric coupling. This manuscript could be considered for publication. However, the specific comments for revision are as follow:

1.The authors should provide a more insightful explanations for why they chose the $InTlNO_2$ monolayer, which is similar to X_2NO_2 (X = In, Tl) [npj Computational Materials (2022) 8:60]. More experimental discussions should be added to make this study more reasonable.

2. There are some transition states between the three stable phases. To establish the true transition state, it is essential for the authors to confirm the corresponding phonon spectra.

3. The origins of the FM and MAE should be given. What are the differences between the p-orbital magnetism and the traditional d-orbital magnetism? For a comprehensive understanding of the magnetic mechanism, it is crucial to incorporate details about the microscopic mechanisms of ferromagnetic coupling. The schematic diagram illustrating magnetic exchange, orbital splitting, and electron hopping paths should be included to elucidate the origin of high Curie temperatures? The origin of the MAE is also important, and please refer to [Results Phys. 53 (2023) 106988].

Reviewer: 2

Comments to the Author

The manuscript introduced Janus 2D multiferroic MXene-analogous oxynitrides, particularly focusing on $InTlNO_2$, with notable multiferroic properties. The authors identified a novel phase (p3) alongside two

original phases (p1 and p2), where all displayed multiferroic behavior with a Curie temperature surpassing room temperature. This work is new and interesting for JPCL. A revision is required before acceptance:

1.The electric polarization may be influenced by both the Janus design and the ferroelectricity. The authors should provide the electric polarization values for p1, p2 and p3.

2.The authors needs to illustrate the possibility for these MXene-analogous oxynitrides to be experimentally fabricated.

3.This manuscript only discussed one compound (InTlNO2) to clarify the 2D asymmetric multiferroics. How to claim the “universal design”?

Reviewer: 3

Comments to the Author

In this manuscript, the authors report their first-principles calculations on a Janus two-dimensional (2D) material, InTlNO2, designed by substituting one layer of In (Tl) ions of In2NO2 (Tl2NO2) withTl (In) ions. The stability of this newly-designed 2D material was verified from phonon spectra and molecular dynamics simulations. Their calculations revealed that the InTlNO2 exhibits three multiferroric phases, denoted as p1, p2 and p3, with Curie temperatures above the room temperature. The activation energy barriers between these phase were evaluated by the nudged elastic band method. Interestingly, p1 favors in-plane and p2 favors out-of-plane easy axis, suggesting a potential approach for tuning the magnetic anisotropy by reversing the out-plane electric polarization. I have the following concerns that should be addressed before I recommend publication.

(1) P1 and p2 exhibit distinct magnetic anisotropy. What’s the underlying origin?

(2) The feasibility and potential approaches of reversing the out-plane electric polarization should be discussed.

(3) The authors stated that “The lattice constant of p3 also lies appropriately between those of p1 and p2, suggesting it may be a transition state.” (line 15-16 of Page 5). However, both the phonon spectrum manifests the dynamics stability of the p3 phase. So it is inappropriate to classify p3 as a transition state.

(4) I suggest the authors to present the configurations of the transition states along the NEB path in Fig. 3.

Author's Response to Peer Review Comments:

Point-to-point response to Reviewers’ comments

Response to the first referee's comments

Reviewer: 1

Recommendation: This paper may be publishable, but major revision is needed; I would like to be invited to review any future revision.

Comments:

Yu et al. proposed a novel Janus 2D multiferroic MXene-analogous oxynitrides ($InTlNO_2$) based on the first-principles calculations. This Janus structure is different from the previous Janus monolayers, such as MoSSe. Three inequivalent phases can be found, exhibiting potential magnetoelectric coupling. This manuscript could be considered for publication. However, the specific comments for revision are as follow:

1.The authors should provide a more insightful explanations for why they chose the $InTlNO_2$ monolayer, which is similar to X_2NO_2 (X = In, Tl) [npj Computational Materials (2022) 8:60]. More experimental discussions should be added to make this study more reasonable.

Response: Thanks for the comments. $InTlNO_2$ and X_2NO_2 (X = In, Tl) share similar structures and properties, which are both p-orbital multiferroics. But the core problems solved by these two designs are different. What X_2NO_2 (X = In, Tl) solved is the long-term problem that ferromagnetism and ferroelectricity can hardly coexist, where the introduction of p-orbital magnetism is a key point. However, they are still type-I multiferroics, where the small magnetoelectric coupling hinders its real application. So, our design aims to give a universal strategy that can improve the magnetoelectric coupling in the type-I multiferroics. By the "twodimensional asymmetric multiferroics" strategy, the electric control of Curie Temperature and magnetic anisotropy can both be easily achieved in $InTlNO_2$, which is absent in traditional type-I multiferroics like X_2NO_2 (X = In, Tl). In a brief, from the structural perspective, there is only one-layer substitution between In and Tl from X_2NO_2 (X = In, Tl) to $InTlNO_2$. But from the physical and functional aspects, there are dramatical difference between X_2NO_2 (X = In, Tl) and $InTlNO_2$. For the experimental consideration, the one-layer substitution will definitely make the synthesis of the Janus $InTlNO_2$ more difficult than that of X_2NO_2 (X = In, Tl). Here, three different phases (zigzag, parallel, armchair) (**Figure R1-1**) that may compete with our $InTlNO_2$ during the fabrication process are considered. Not surprisingly, the DFT-calculated energies (Table R1) are almost same as that of p1 $InTlNO_2$. This will undoubtedly create difficulties in the fabrication. Therefore, we proposed a three-step method that may accurately synthesize the Janus $InTlNO_2$ based on the previous successful examples. [1, 2] First step is the fabrication of In_2N (Tl_2N). Given the successful synthesis of two-dimensional titanium nitride Ti_4N_3 (MXene)[2] since 2016, the fabrication of In_2N (Tl_2N) would not be too difficult to be achieved for experimentalists and similar molten salt treatment may be helpful. Second step is the one-layer substitution that transforms In_2N (Tl_2N) into InTlN. The successful synthesis of MoSSe can be taken for an important reference, where there are also two different approaches to achieve the Janus design.[1] One method used hydrogen plasma to strip off the top-S atoms of MoS_2 and replace them with hydrogen atoms, forming MoSH.[3] Then the plasma was switched off and the loaded Se powder was then thermally vaporized to replace the top layer H atoms with Se atoms, forming stable Janus MoSSe.[3] The method may be applied to In_2N (Tl_2N) if there are suitable Tl

(In) resources. The other method to obtain the Janus MoSSe in a typical setup was through the direct sulfurization of top layer Se under specific temperature, which is 800 °C in this situation.[4] While temperature lower than 750 °C or higher than 850 °C is harmful to the fabrication of Janus MoSSe.[4] Therefore, it is inspired that the one-layer substitution of In_2N (Tl_2N) may be also achieved by finely control of the reaction temperature. The last step is to terminate the obtained Janus InTlN with functional groups which is oxygen atoms here. The functionalization should naturally occur in the ambient condition, after which the Janus $InTlNO_2$ can be obtained.

Table R1. Relative energy of different $InTlNO_2$ (the energy of p1 is set to 0).

Phase	p1	p2	p3	zigzag	parallel	armchair
Relative energy (meV/atom)	0	12	4	-1	-2	-3

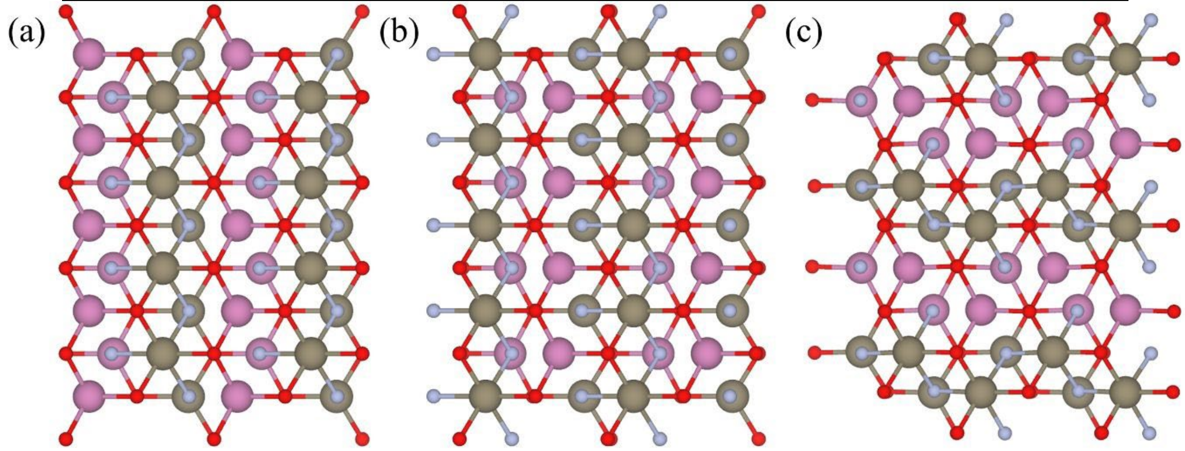

Figure R1-1. Three potential competing phases of $InTlNO_2$: (a) zigzag, (b) parallel and (c) armchair.

The following sentences were updated in our revised manuscript:

"Although $InTlNO_2$ is similar to the parent structure X_2NO_2 (X = In, Tl), X_2NO_2 (X = In, Tl) provide the p-orbital multiferroics for the incompatibility between the d-orbital magnetism and d^0 ferroelectricity in traditional multiferroics, while $InTlNO_2$ for enhanced magnetoelectric coupling. Our strategy may pave the way for the incompatibility between prominent magnetoelectric coupling and considerable polarization." (Page 10, Lines 11-16)

"To make our design more reasonable, we compare the energies of three different phases (**Figure S20**) that may compete with the Janus $InTlNO_2$ during the fabrication and find their energies are relatively similar (**Table S2**). Therefore, a three-step method is proposed that may accurately synthesize the Janus $InTlNO_2$ based on the previous successful examples.[1, 2] First step is the fabrication of In_2N (Tl_2N). Given the successful synthesis of two-dimensional titanium nitride Ti_4N_3 (MXene)[2] since 2016, the fabrication of In_2N (Tl_2N) can use similar molten salt treatment. Second step is the one-layer substitution that transforms In_2N (Tl_2N) into InTlN, which may be achieved by either hydrogen plasma assisted substitution[3] or finely control of the temperature during the substituting process[4]. The last

step is to terminate the obtained pure InTlN with functional groups which is oxygen atoms here. The functionalization should naturally occur in the ambient condition, after which the Janus $InTlNO_2$ can be obtained." (Page 10, Lines 18-29)

2. There are some transition states between the three stable phases. To establish the true transition state, it is essential for the authors to confirm the corresponding phonon spectra. **Response:** Thanks for the comments. The phonon spectra of corresponding transition states in **Figure 3** are calculated and shown below (**Figure R1-2**). The transition state between p1 and p3 is called p13 and that between p2 and p3 is called p23. The imaginary modes are found around Γ point (red dashed box) for both p13 and p23, confirming their transition-state nature.

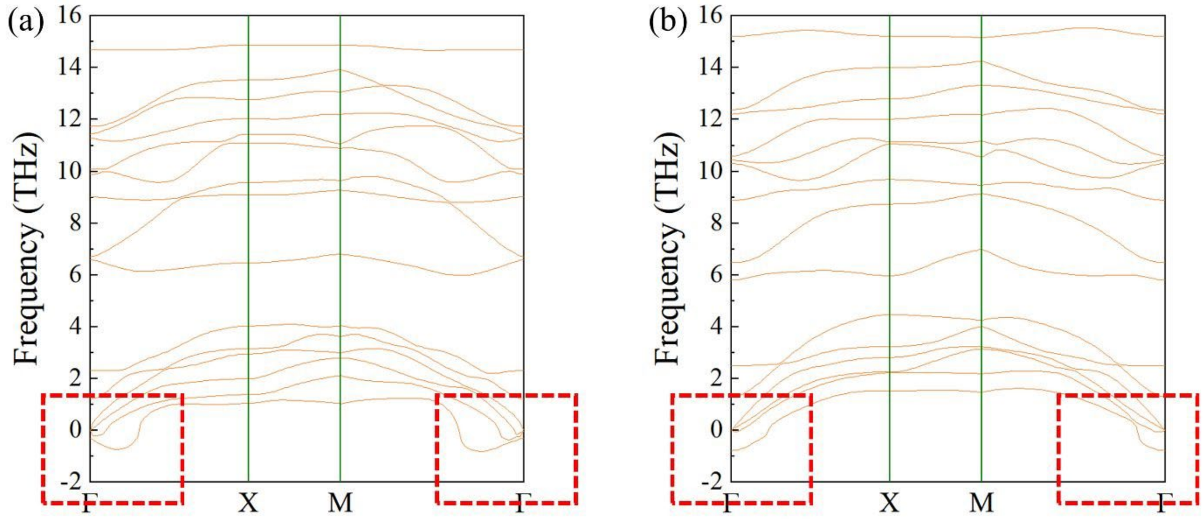

Figure R1-2. The phonon spectra of (a) p12 and (b) p23 (The imaginary modes are marked by red dashed box).

The following sentences were updated in our revised manuscript:

"The evident imaginary modes in the phonon dispersions of the transition states (**Figure S7**) indicate their instability." (Page 8, Lines 1-2)

3. The origins of the FM and MAE should be given. What are the differences between the porbital magnetism and the traditional d-orbital magnetism? For a comprehensive understanding of the magnetic mechanism, it is crucial to incorporate details about the microscopic mechanisms of ferromagnetic coupling. The schematic diagram illustrating magnetic exchange, orbital splitting, and electron hopping paths should be included to elucidate the origin of high Curie temperatures? The origin of the MAE is also important, and please refer to [Results Phys. 53 (2023) 106988].

Response: Thanks for the comments. In the current multiferroics research, magnetism is exclusively established in the paradigm of ordered spins of electrons in the partially occupied d orbitals of transition metal atoms, while ferroelectricity is usually related to the residual polarization due to off-centered ions with empty d orbitals,[5] which poses an outstanding challenge for the field of multiferroics.[6] To solve this

problem, p-orbital magnetism is promising since it is compatible with ferroelectricity. For the microscopic mechanisms of ferromagnetic coupling, p1 and p2 are similar since they are both half-metallic, while p3 is different from the other two due to its semiconducting nature. For p1 and p2, the ferromagnetic coupling can be attributed to the Stoner effect. According to the Stoner model, the itinerant ferromagnetism occurs if $J\,D(E_F) > 1$ is satisfied, where $D(E_F)$ is the DOS at the Fermi energy E_F, and J denotes the strength of the exchange interaction.[7] It is found that the $D(E_F)$ are 9.52 and 3.78 states/eV for p1 and p2, respectively (**Figure R1-3**), while J is about 2.5 eV. The value of J is a little larger than the previous reports because of the use of HSE06 functional in our calculations. So, both meet the Stoner criterion for a stable ferromagnetic state. Moreover, the half-metallic characteristics also indicate the carrier-mediated double exchange is responsible for the ferromagnetic couplings in both phases.[7-9] For p3, all the p orbitals of both O and N ions are hybridized extensively, it is difficult to give a schematic diagram illustrating the exchange splitting. Therefore, the strong ferromagnetic coupling and high Curie Temperature of p3 can be attributed to the extended direct p-p interaction, similar to the scenario of single layer SiN and PN_2.[6, 10] Moreover, the nearest N-In-N bonding angle is close to 90° (**Figure R1-4**), which can also contribute to the ferromagnetic coupling of p3 according to the Goodenough-Kanamori-Anderson (GKA) rules of the super exchange theorem.[11] For the origin of magnetic anisotropic energy (MAE), the literature mentioned **[Results Phys. 53 (2023) 106988]** has been taken for a critical reference. The magnetic anisotropies of p1 and p2 are attributed to d orbitals of In and Tl. The contribution of MAE of each orbital can be obtained by the orbital-resolved ΔE_{soc}, where positive and negative values represent out-of-plane and in-plane magnetic anisotropy, respectively. The interactions between d orbitals of In and Tl ions contribute most to the total magnetic anisotropy (**Figures R1-5 & R1-6**), while those of p orbitals and other ions are negligible. For p1, the interactions between the d_{xz} and d_{yz} orbitals of Tl ions and d_{xy} and $d_{x^2-y^2}$ orbitals of In ions contribute to the in-plane magnetic anisotropy. For p2, the magnetic anisotropy is dominated by the interaction among the Tl ions' d orbitals. However, the interactions between the d_{yz} and d_{z^2} (d_{xy} and $d_{x^2-y^2}$) are stronger than that between the d_{xz} and d_{yz} orbitals, rendering p2 the out-of-plane magnetic anisotropy. For p3, the d orbitals of Tl ions contribute little because the interactions counteract with each other, while the p orbitals of both Tl and In ions favor the in-plane magnetic anisotropy (**Figures R1-7 & R1-8**). The interactions between the d and p orbitals of In ions should account for the deviation of the magnetic anisotropy from in-plane direction (**Figures R1-9 & R1-10**).

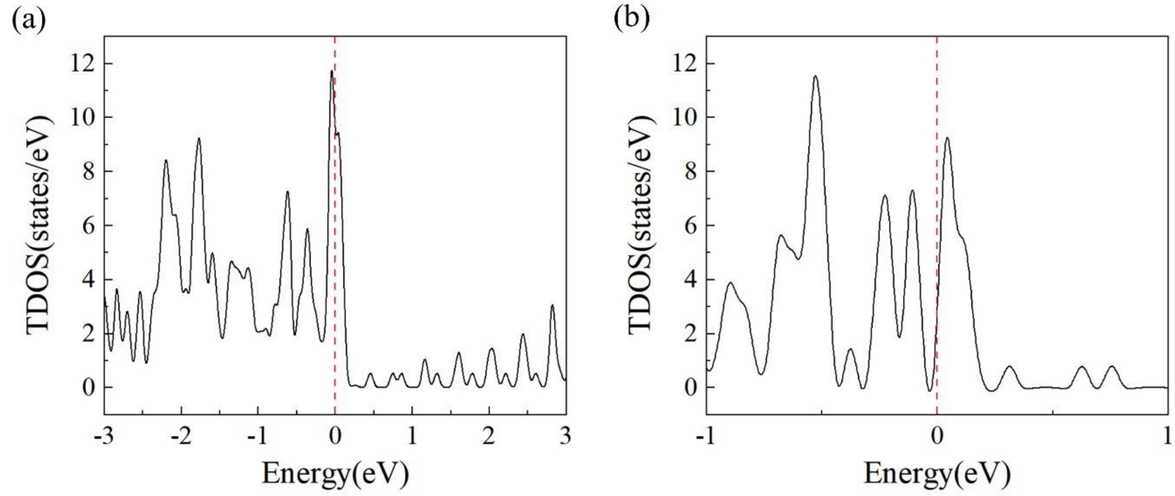

Figure R1-3. Non-spin polarized TDOS of (a) p1 and (b) p2.

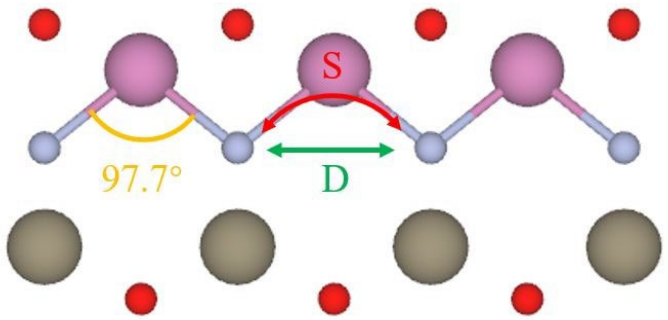

Figure R1-4. A schematic diagram of magnetic exchange interaction in p3 (D: direct exchange, S: super exchange).

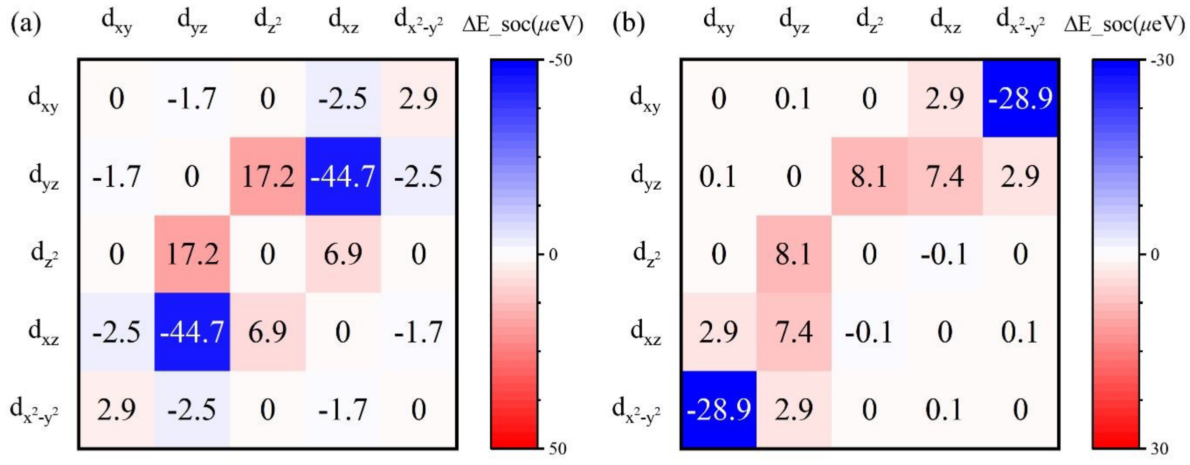

Figure R1-5. d-orbital-resolved ΔE_{soc} for p1: (a) Tl and (b) In.

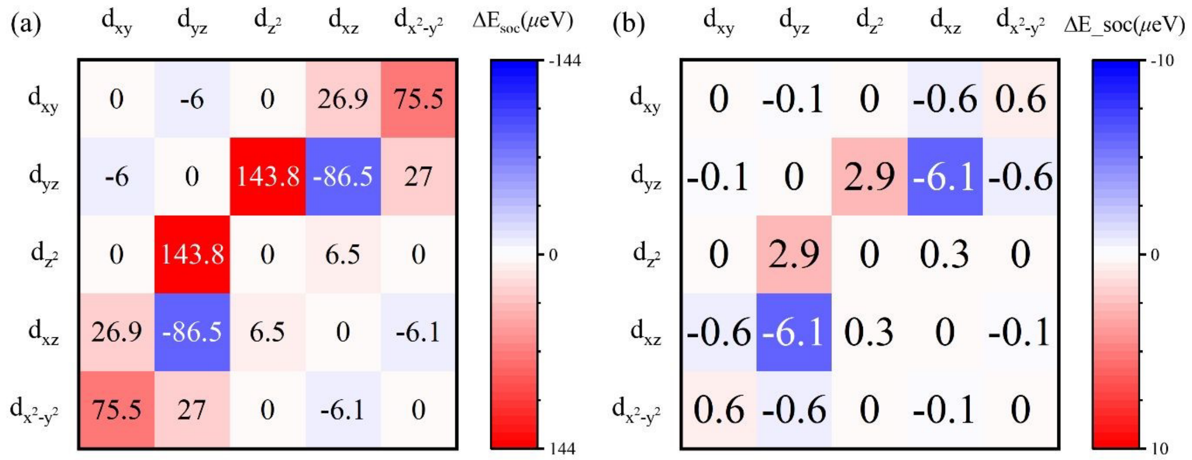

Figure R1-6. d-orbital-resolved ΔE_{soc} for p2: (a) Tl and (b) In.

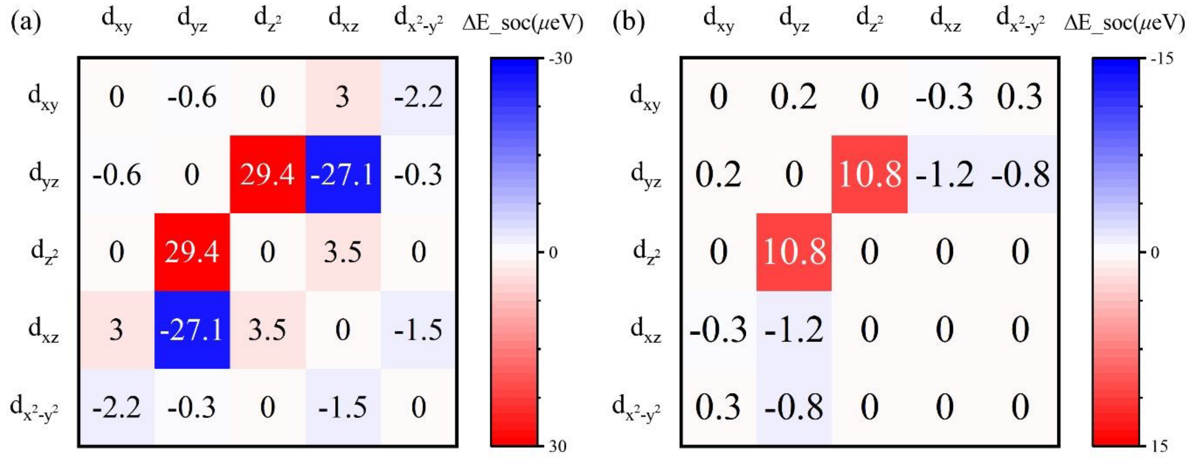

Figure R1-7. d-orbital-resolved ΔE_{soc} for p3: (a) Tl and (b) In.

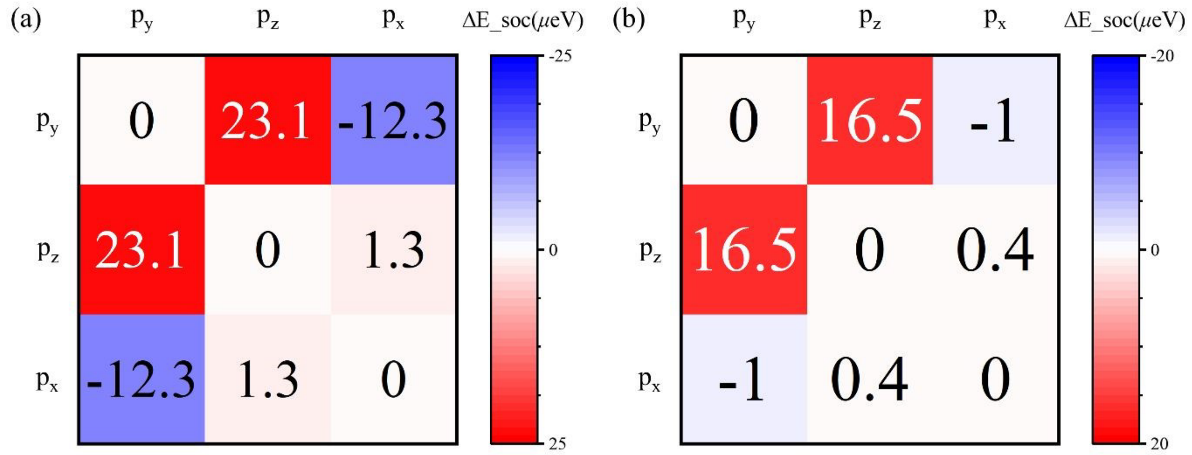

Figure R1-8. p-orbital-resolved ΔE_{soc} for p3: (a) Tl and (b) In.

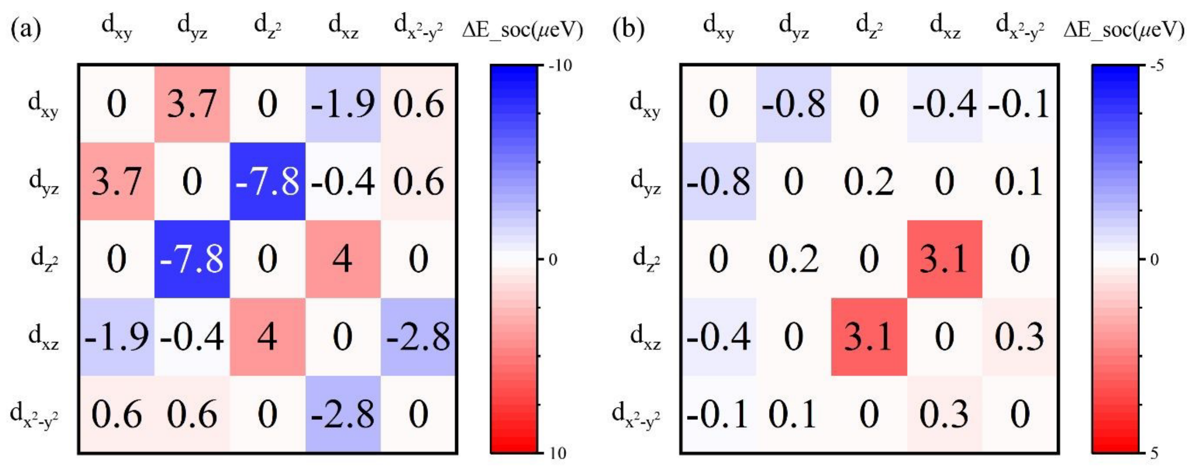

Figure R1-9. d-orbital-resolved ΔE_{soc} (deviation between in-plane direction and easy axis) of for p3: (a) Tl and (b) In (Positive values favor the easy axis, while negative values favor the inplane direction).

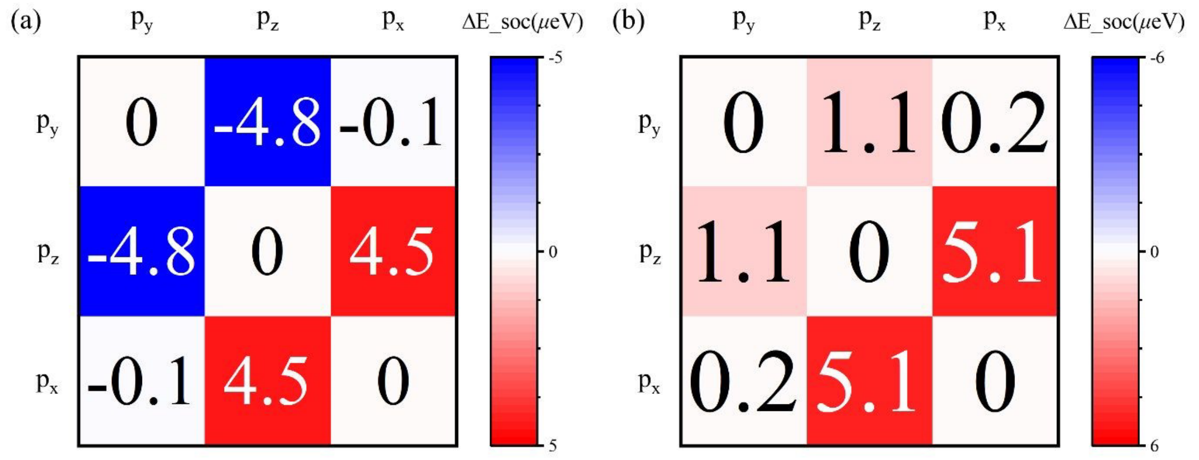

Figure R1-10. p-orbital-resolved ΔE_{soc} (deviation between the in-plane direction and easy axis) for p3: (a) Tl and (b) In (Positive values favor the easy axis, while negative values favor the inplane direction).

The following sentences were updated in our revised manuscript:

"For p1 and p2, the ferromagnetic coupling can be explained by the Stoner effect. According to the Stoner model, the itinerant ferromagnetism occurs if $J\,D(E_F) > 1$ is satisfied, where $D(E_F)$ is the DOS at the Fermi energy E_F, and J denotes the strength of the exchange interaction.[7] $D(E_F)$ are 9.52 and 3.78 states/eV for p1 and p2, respectively (**Figure R1-3**), while J is about 2.5 eV. The value of J is slightly larger than the previous reports because of the use of HSE06 functional in our calculations." (Page 6, Lines 24-29)

"Because of the semiconducting nature, the long-range magnetic coupling between the moments in the p3 phase can be attributed to the direct p-p interactions.[6, 10, 12] Moreover, the nearest N-In-N bonding angle is close to 90° (**Figure S6**), resulting in the super exchange for the ferromagnetic coupling of p3 according to the Goodenough-Kanamori-Anderson (GKA) rules.[11]" (Page 7, Lines 13-17)

“The magnetic anisotropies of p1 and p2 are attributed to the d orbitals of In and Tl. The orbitalresolved ΔE_{soc} can be used to identify the contribution, where positive and negative values represent out-of-plane and in-plane magnetic anisotropy, respectively.[13] The interactions between the d orbitals of In and Tl ions contribute most to the total magnetic anisotropy (**Figures S13 & S14**), while those of p orbitals and other ions are negligible. For p1, the interactions between the d_{xz} and d_{yz} orbitals of Tl ions and d_{xy} and $d_{x^2-y^2}$ orbitals of In ions contribute to the in-plane magnetic anisotropy. For p2, the magnetic anisotropy is dominated by the interaction among the Tl ions’ d orbitals. However, the interactions between the d_{yz} and d_{z^2} (d_{xy} and $d_{x^2-y^2}$) are stronger than that between the d_{xz} and d_{yz} orbitals, rendering p2 the outof-plane magnetic anisotropy.” (Page 9, Lines 24-34)

“p3 shows relatively weak magnetic anisotropy with a MAE of 40 μeV (**Figure S15**), whose easy-axis lies on the yz plane with an angle of 116° to the z axis. We find that p3 favors inplane direction due to the interactions between the p orbitals of both Tl and In ions (**Figures S16 & S17**), while the deviated easy axis from the in-plane direction is originated from the interactions between In ions’ p_x and p_z orbitals (**Figures S18 & S19**).” (Page 9, Line 34; Page 10, Lines 1-5)

Response to the second referee's comments

Reviewer: 2

Recommendation: This paper is publishable subject to minor revisions noted. Further review is not needed.

Comments:

The manuscript introduced Janus 2D multiferroic MXene-analogous oxynitrides, particularly focusing on $InTlNO_2$, with notable multiferroic properties. The authors identified a novel phase (p3) alongside two original phases (p1 and p2), where all displayed multiferroic behavior with a Curie temperature surpassing room temperature. This work is new and interesting for JPCL. A revision is required before acceptance:

1.The electric polarization may be influenced by both the Janus design and the ferroelectricity. The authors should provide the electric polarization values for p1, p2 and p3.

Response: Thanks for the comments. Since both p1 and p2 are metallic, it is difficult for them to get the polarization values directly based on the Berry phase method. Here, we calculated the polarizations based on a two-step method proposed in the previous reports.[14, 15] First, we obtained the vacuum level difference (Δ□) by electrostatic potential calculations (**Figure R21**). Then, the electric dipole moment was derived through the parallel-plate-capacitor model. Finally, the polarization values for p1, p2 and p3 were obtained as 2.30 pC/m, -17.97pC/m and -4.43 pC/m, respectively, all comparable with those of X_2NO_2 (X = In, Tl).

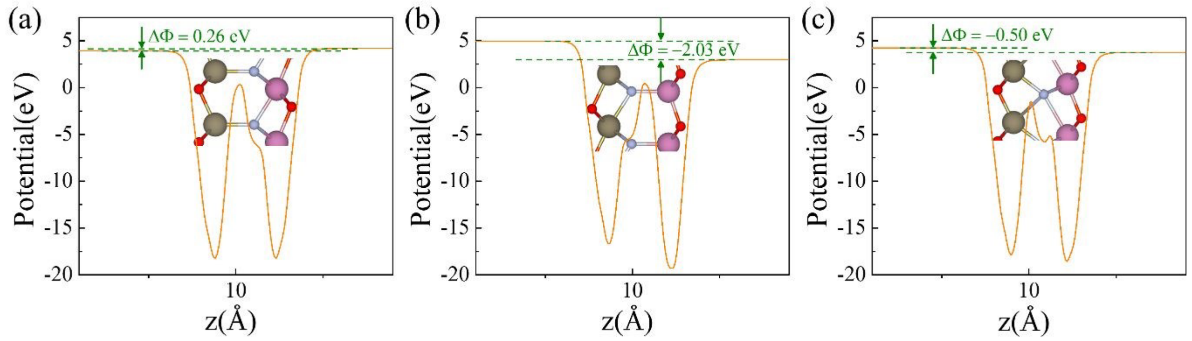

Figure R2-1. Electrostatic potential of p1, p2 and p3.

The following sentences were updated in our revised manuscript:

"To check whether the transformation under external field is possible, the electric polarization of $InTlNO_2$ was investigated based on a reported method.[14, 15] The calculated vacuum level differences (□□) are 0.26, -2.03 and -0.50 eV, and hence the out-of-plane polarizations are 2.30, -17.97 and -4.43 pC/m for p1, p2 and p3, respectively. Therefore, we can conclude that different phases of $InTlNO_2$ exhibit different out-of-plane polarizations, which makes the phase transition among p1, p2 and p3 viable by the external electric field." (Page 8, Lines 18-24)

2.The authors need to illustrate the possibility for these MXene-analogous oxynitrides to be experimentally fabricated.

Response: Thanks for the comments. The experimental fabrication of these MXene-analogous oxynitrides can be divided into three parts. First step is the fabrication of In_2N (Tl_2N). Given the successful synthesis of two-dimensional titanium nitride Ti_4N_3 (MXene)[2] since 2016, the fabrication of In_2N (Tl_2N) 'would not be too difficult to be achieved for experimentalists and similar molten salt treatment may be helpful. Second step is the one-layer substitution that transforms In_2N (Tl_2N) into InTlN. The successful synthesis of MoSSe can be taken for an important reference, where there are also two different approaches to achieve the Janus design.[1] One method used hydrogen plasma to strip off the top-S atoms of MoS_2 and replace them with hydrogen atoms, forming MoSH.[3] Then the plasma was switched off and the loaded Se powder was then thermally vaporized to replace the top layer H atoms with Se atoms, forming stable Janus MoSSe.[3] The method may be applied to In_2N (Tl_2N) if there are suitable Tl (In) resources. The other method to obtain the Janus MoSSe in a typical setup was through the direct sulfurization of top layer Se under specific temperature, which is 800 °C in this situation.[4] While temperature lower than 750 °C or higher than 850 °C is harmful to the fabrication of Janus MoSSe.[4] Therefore, it is inspired that the one-layer substitution of In_2N (Tl_2N) may be also achieved by finely control of the reaction temperature. The last step is to terminate the obtained pure In_2N (Tl_2N) with functional groups which is oxygen atoms here. The functionalization should naturally occur in the ambient condition, after which the Janus $InTlNO_2$ can be obtained.

The following sentences were updated in our revised manuscript:

"To make our design more reasonable, we compare the energies of three different phases (**Figure S20**) that may compete with the Janus $InTlNO_2$ during the fabrication and find their energies are relatively similar (**Table S2**). Therefore, a three-step method is proposed that may accurately synthesize the Janus $InTlNO_2$ based on the previous successful examples.[1, 2] First step is the fabrication of In_2N (Tl_2N). Given the successful synthesis of two-dimensional titanium nitride Ti_4N_3 (MXene)[2] since 2016, the fabrication of In_2N (Tl_2N) can use similar molten salt treatment. Second step is the one-layer substitution that transforms In_2N (Tl_2N) into InTlN, which may be achieved by either hydrogen plasma assisted substitution[3] or finely control of the temperature during the substituting process[4]. The last step is to terminate the obtained pure InTlN with functional groups which is oxygen atoms here. The functionalization should naturally occur in the ambient condition, after which the Janus $InTlNO_2$ can be obtained." (Page 10, Lines 18-29)

3.This manuscript only discussed one compound ($InTlNO_2$) to clarify the 2D asymmetric multiferroics. How to claim the "universal design"?

Response: Thanks for the comments. The "universal design" here means that if there are two distinct 2D multiferroic materials sharing similar structures like In_2NO_2 and Tl_2NO_2, then we can directly have the 2D asymmetric multiferroic phases with strong magnetoelectric coupling by the one-layer substitution. The stability is another key point for consideration. It will be important for theoretical and experimental efforts searching for 2D multiferroics.

Response to the third referee's comments

Reviewer: 3

Recommendation: This paper may be publishable, but major revision is needed; I would like to be invited to review any future revision.

Comments:

In this manuscript, the authors report their first-principles calculations on a Janus twodimensional (2D) material, $InTlNO_2$, designed by substituting one layer of In (Tl) ions of In_2NO_2 (Tl_2NO_2) with Tl (In) ions. The stability of this newly-designed 2D material was verified from phonon spectra and molecular dynamics simulations. Their calculations revealed that the $InTlNO_2$ exhibits three multiferroic phases, denoted as p1, p2 and p3, with Curie temperatures above the room temperature. The activation energy barriers between these phases were evaluated by the nudged elastic band method. Interestingly, p1 favors in-plane and p2 favors out-of-plane easy axis, suggesting a potential approach for tuning the magnetic anisotropy by reversing the out-plane electric polarization. I have the following concerns that should be addressed before I recommend publication.

(1) P1 and p2 exhibit distinct magnetic anisotropy. What's the underlying origin?

Response: Thanks for the comments. The magnetic anisotropies of p1 and p2 are attributed to d orbitals of In and Tl. The contribution of MAE of each orbital can be obtained by the orbitalresolved ΔE_{soc}, where positive and negative values represent out-of-plane and in-plane magnetic anisotropy, respectively. The interactions between d orbitals of In and Tl ions contribute most to the total magnetic anisotropy (**Figures R3-1 & R3-2**), while those of p orbitals and other ions are negligible. For p1, the interactions between the d_{xz} and d_{yz} orbitals of Tl ions and d_{xy} and $d_{x^2-y^2}$ orbitals of In ions contribute to the in-plane magnetic anisotropy. For p2, the magnetic anisotropy is dominated by the interaction among the Tl ions' d orbitals. However, the interactions between the d_{yz} and d_{z^2} (d_{xy} and $d_{x^2-y^2}$) are stronger than that between the d_{xz} and d_{yz} orbitals, rendering p2 the out-of-plane magnetic anisotropy.

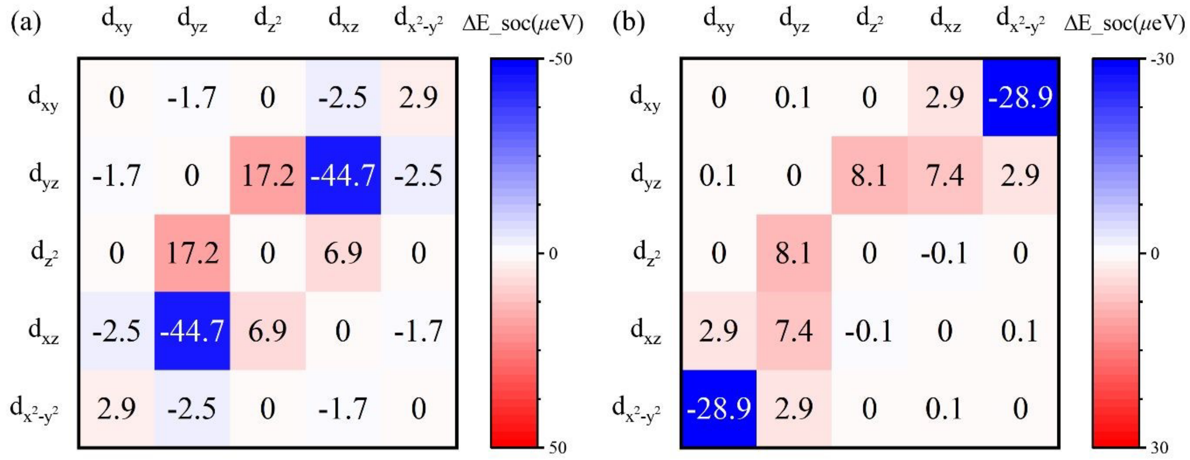

Figure R3-1. Orbital-resolved ΔE_{soc} of d orbitals for p1: (a) Tl and (b) In.

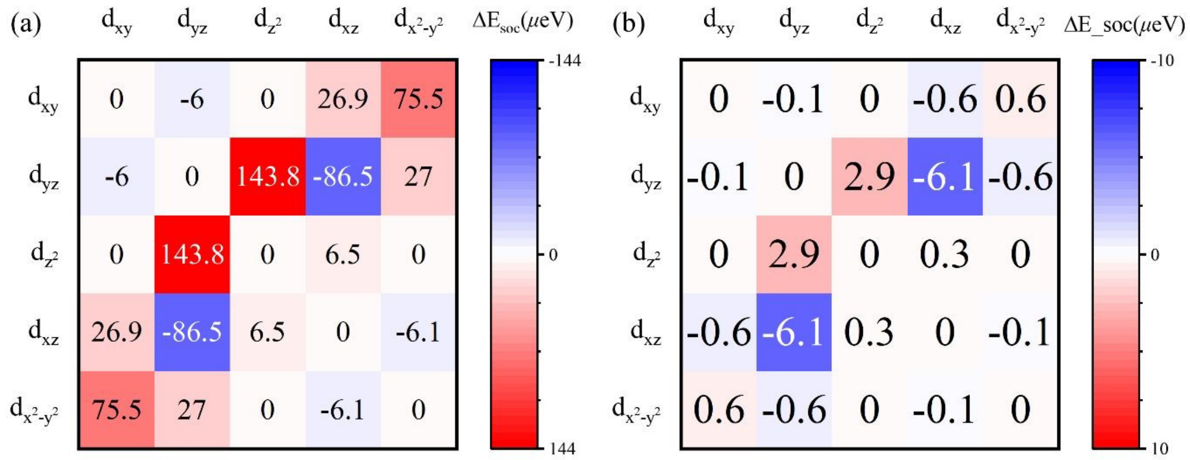

(a)	d_{xy}	d_{yz}	d_{z^2}	d_{xz}	$d_{x^2-y^2}$
d_{xy}	0	-6	0	26.9	75.5
d_{yz}	-6	0	143.8	-86.5	27
d_{z^2}	0	143.8	0	6.5	0
d_{xz}	26.9	-86.5	6.5	0	-6.1
$d_{x^2-y^2}$	75.5	27	0	-6.1	0

(b)	d_{xy}	d_{yz}	d_{z^2}	d_{xz}	$d_{x^2-y^2}$
d_{xy}	0	-0.1	0	-0.6	0.6
d_{yz}	-0.1	0	2.9	-6.1	-0.6
d_{z^2}	0	2.9	0	0.3	0
d_{xz}	-0.6	-6.1	0.3	0	-0.1
$d_{x^2-y^2}$	0.6	-0.6	0	-0.1	0

Figure R3-2. Orbital-resolved ΔE_{soc} of d orbitals for p2: (a) Tl and (b) In.

The following sentences were updated in our revised manuscript:

"The magnetic anisotropies of p1 and p2 are attributed to the d orbitals of In and Tl. The orbitalresolved ΔE_{soc} can be used to identify the contribution, where positive and negative values represent out-of-plane and in-plane magnetic anisotropy, respectively.[13] The interactions between the d orbitals of In and Tl ions contribute most to the total magnetic anisotropy (**Figures S13 & S14**), while those of p orbitals and other ions are negligible. For p1, the interactions between the d_{xz} and d_{yz} orbitals of Tl ions and d_{xy} and $d_{x^2-y^2}$ orbitals of In ions contribute to the in-plane magnetic anisotropy. For p2, the magnetic anisotropy is dominated by the interaction among the Tl ions' d orbitals. However, the interactions between the d_{yz} and d_{z^2} (d_{xy} and $d_{x^2-y^2}$) are stronger than that between the d_{xz} and d_{yz} orbitals, rendering p2 the outof-plane magnetic anisotropy." (Page 9, Lines 24-34)

(2) The feasibility and potential approaches of reversing the out-plane electric polarization should be discussed.

Response: Thanks for the comments. Traditionally, the out-of-plane electric polarization of ferroelectric material can be reversed by an external electric field. Therefore, if the three distinct phases (p1, p2 and p3) exhibit different values for the out-of-plane electric polarization, it can be switched by a perpendicular electric field. To validate this point, we calculated the polarization values of p1, p2 and p3. Since both p1 and p2 are metallic, it is difficult for them to get the polarization directly based on the Berry phase method. Here, we calculated the polarizations based on a two-step method proposed in the previous reports.[14, 15] First, we obtained the vacuum level difference (Δ□) by the electrostatic potential calculations (**Figure R3-3**). Then, the electric dipole moment was derived through the parallel-plate-capacitor model. The polarization values for p1, p2 and p3 were obtained as 2.30 pC/m, -17.97 pC/m and -4.43 pC/m, respectively, which are comparable with those of X_2NO_2 (X = In, Tl). Therefore, we can conclude that the different phases of $InTlNO_2$ exhibit different out-of-plane electric polarizations, which make the phase transition among p1, p2 and p3 possible by an external electric field. Another issue is the exact value of the electric field required to switch the outof-plane polarization. Since the quantitative calculations for reversing electric field is difficult in VASP, a

qualitative analysis based on the CI-NEB calculated energy barriers and electric polarization values is given here. Due to the asymmetric energy barriers, six kinds of transitions were considered (p1 to p2, p2 to p1, p1 to p3, p3 to p1, p2 to p3, and p3 to p2). According to the CI-NEB curve and the calculated electric polarization, p1 can switch to p2 directly under a large positive electric field, while a medium negative electric field is required to transform p2 back to p1 (**Figure R3-4**). For p1 and p3, small external field can achieve the transformation between them. p1 can directly cross p3 and transform into p2 if the electric field is large enough to overcome the energy barrier between p3 and p2 (**Figure R3-5**). The scenario for p2 and p3 may be a little more complicated (**Figure R3-6**). Relatively large positive electric field is demanded to directly transform p3 to p2, while a negative electric field may always transform p2 to p1 first since the energy barrier for p3 to p1 is much smaller. Therefore, if we want to transform p2 into p3, a negative electric field is required first to switch p2 to p1, and then a smaller positive electric field can transform p1 to p3 (p2→p1→p3). We also considered the effect of strain on the relative energy of different phases, which can influence the transformation (**Figure R3-7**). When the strain is low, the relative energies among three phases are not affected. In the orange region, where the strain is more negative than -3% or between 2% and 5%, p3 becomes the most stable phase. When the tensile strain is larger than 5%, the relative energy between p1 and p2 reversed, while p3 is still the most stable phase. Therefore, we conclude that the strain may have a pronounced effect on the transition of different phases because of its evident influence on the relative energy of p1, p2 and p3.

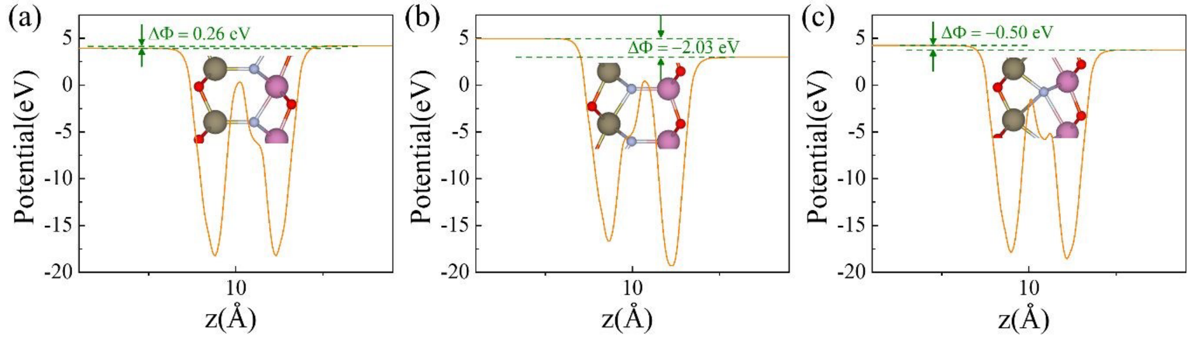

Figure R3-3. Electrostatic potential of p1, p2 and p3.

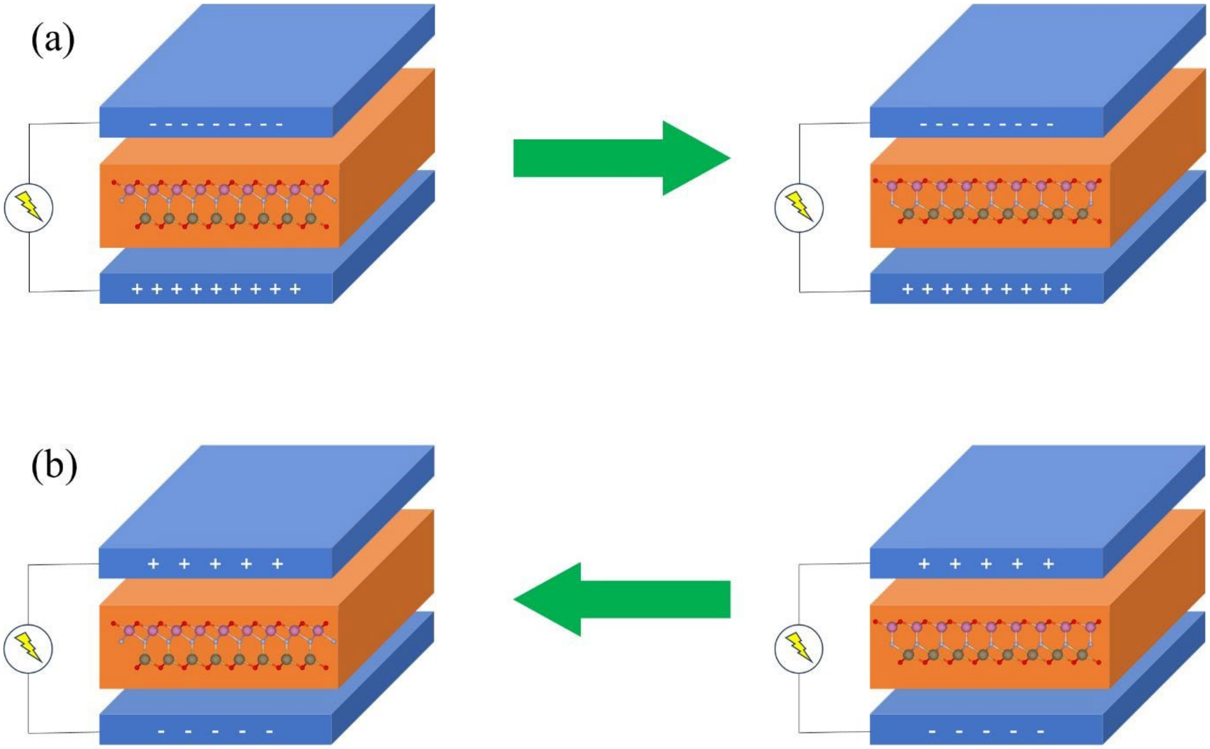

Figure R3-4. (a) p1 to p2 and (b) p2 to p1 (The number of symbols is directly proportional to the electric field strength, same as below).

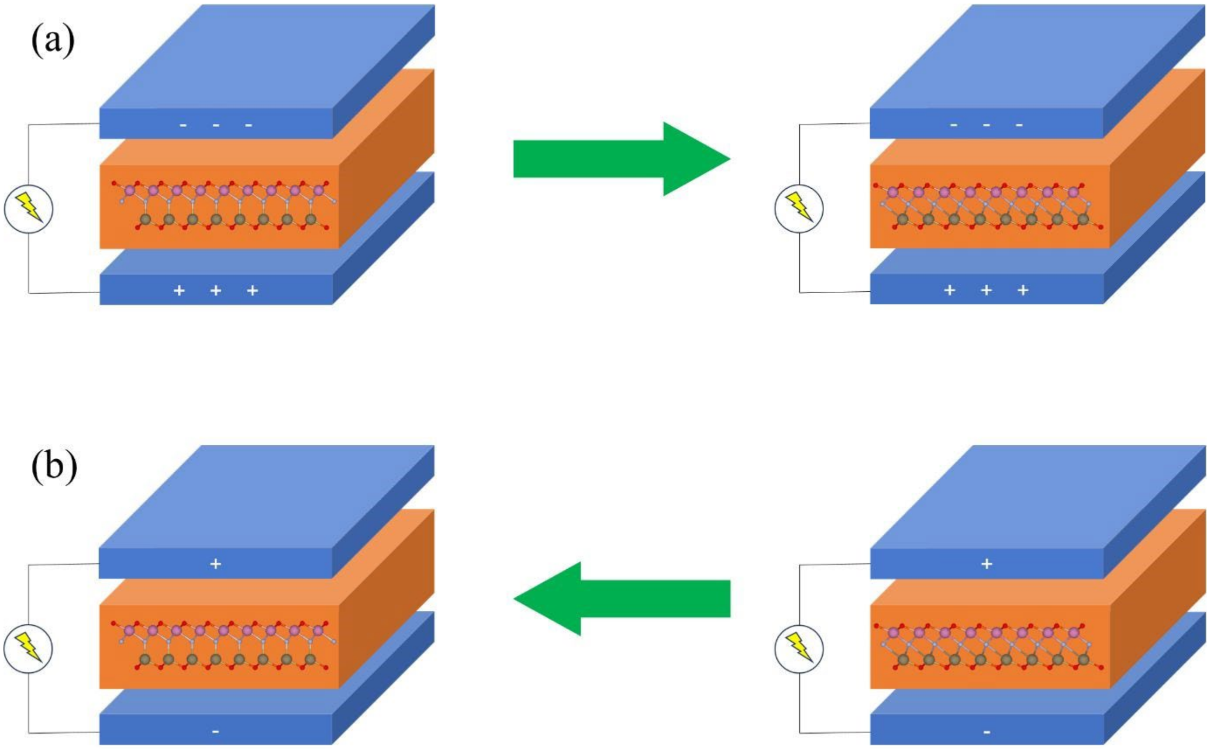

Figure R3-5. (a) p1 to p3 and (b) p3 to p1.

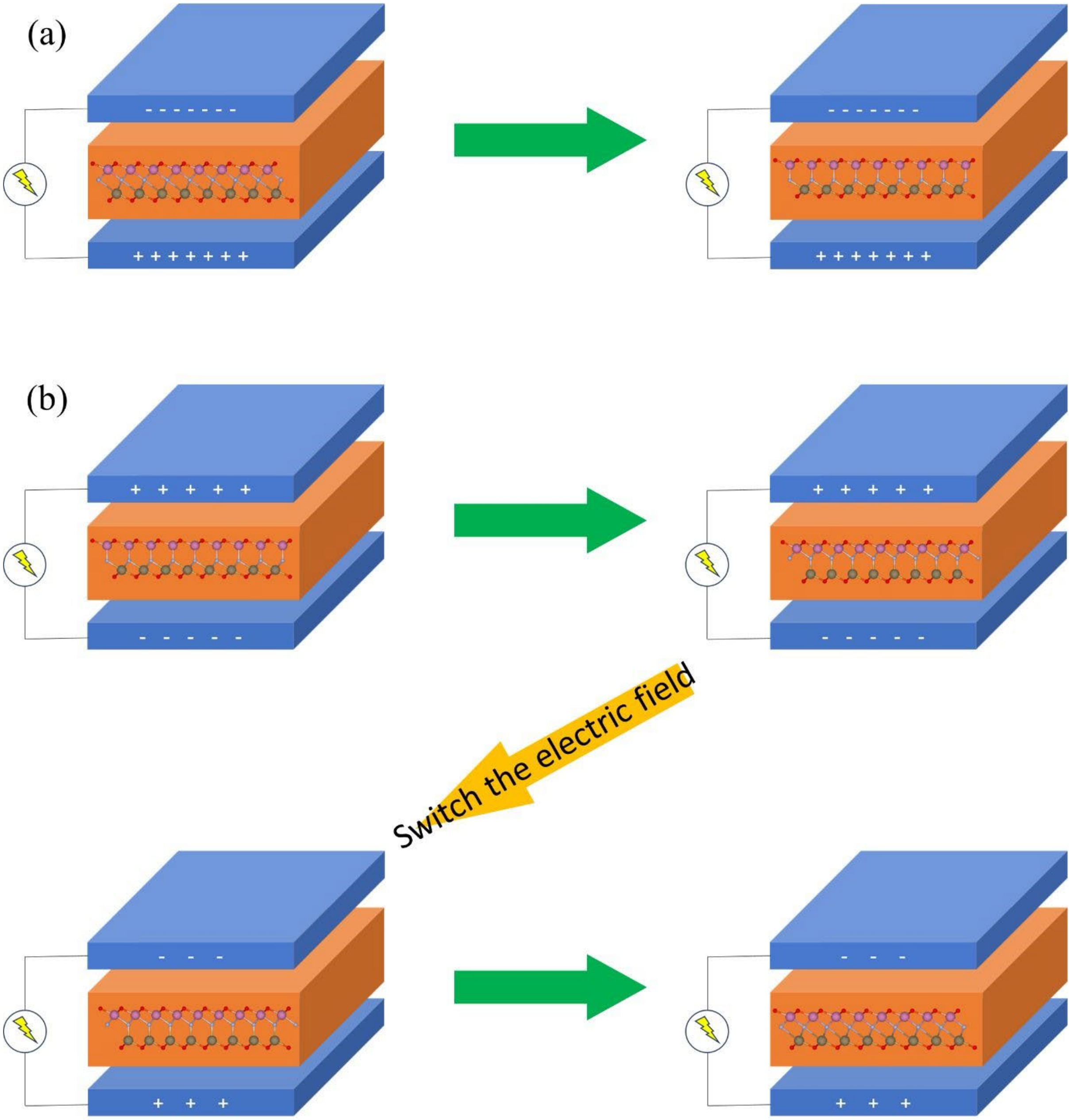

Figure R3-6. (a) p3 to p2 and (b) p2 to p3 (p2→p1→p3).

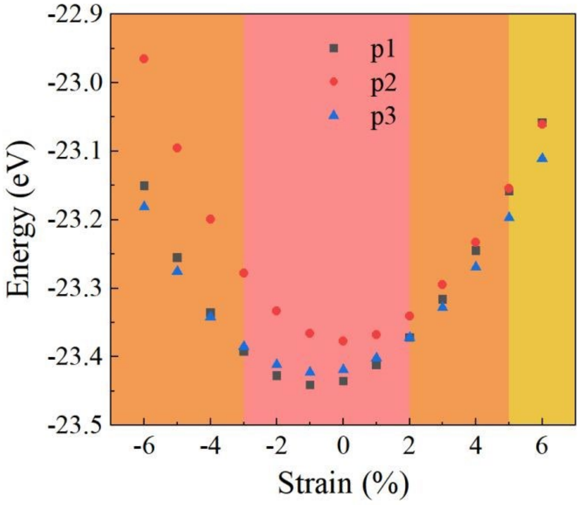

Figure R3-7. Relative energy of p1, p2 and p3 under strain (-6% to 6%).

The following sentences were updated in our revised manuscript:

"To check whether the transformation under external field is possible, the electric polarization of $InTlNO_2$ was investigated based on a reported method.[14, 15] The calculated vacuum level differences (□□) are 0.26, -2.03 and -0.50 eV, and hence the out-of-plane polarizations are 2.30, -17.97 and -4.43 pC/m for p1, p2 and p3, respectively. Therefore, we can conclude that different phases of $InTlNO_2$ exhibit different out-of-plane polarizations, which makes the phase transition among p1, p2 and p3 viable by the external electric field. Meanwhile, due to the asymmetric energy barriers, six kinds of transitions need individual consideration (p1 to p2, p2 to p1, p1 to p3, p3 to p1, p2 to p3 and p3 to p2). A detailed discussion about electric transformation is included in the **Supporting Information (Figures S9-S11)**. We also considered the effect of strain on the relative energy of different phases, which can influence the transformation (**Figure S12**). When the strain is low, the relative energies among three phases are not affected. In the orange region, where the strain is more negative than -3% or between 2% and 5%, p3 becomes the most stable phase. When the tensile strain is larger than 5%, the relative energy between p1 and p2 reversed, while p3 is still the most stable phase. Therefore, we conclude that the strain may have a pronounced effect on the transition of different phases because its evident influence on the relative energy of p1, p2 and p3." (Page 8, Lines 18-35)

The following sentences were updated in our revised Supporting Information:

"Due to the asymmetric energy barriers, six kinds of transitions were considered (p1 to p2, p2 to p1, p1 to p3, p3 to p1, p2 to p3 and p3 to p2). According to the CI-NEB curve and the calculated electric polarization, p1 can switch to p2 directly under a large positive electric field, while a medium negative electric field is required to transform p2 back to p1 (**Figure S9**). For p1 and p3, small external field can achieve the transformation between them. p1 can directly cross p3 and transform into p2 if the electric field is large enough to overcome the energy barrier between p3 and p2 (**Figure S10**). The scenario for p2 and p3 may be a little more complicated (**Figure S11**). Relatively large positive electric field is demanded to directly transform p3 to p2, while a negative electric field may always transform

p2 to p1 first since the energy barrier for p3 to p1 is much smaller. Therefore, if we want to transform p2 into p3, a negative electric field is required first to switch p2 to p1, and then a smaller positive electric field can transform p1 to p3 (p2→p1→p3).” (Page S8, Lines 3-13; Page S9, Line 1)

(3) The authors stated that “The lattice constant of p3 also lies appropriately between those of p1 and p2, suggesting it may be a transition state.” (line 15-16 of Page 5). However, both the phonon spectrum manifests the dynamics stability of the p3 phase. So, it is inappropriate to classify p3 as a transition state.

Response: Thanks for the comments. The relative statements were deleted.

(4) I suggest the authors to present the configurations of the transition states along the NEB path in Fig. 3.

Response: Thanks for the comments. **Figure 3** was updated with both top view (up) and side view (down) included (**Figure R3-8.**).

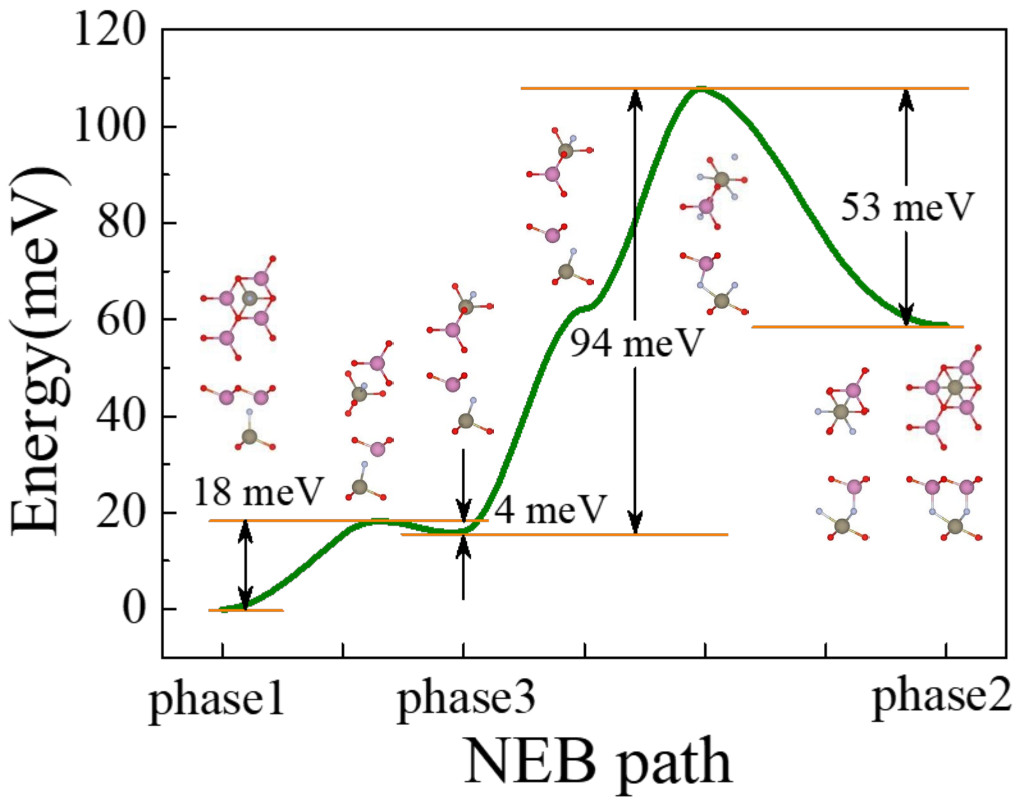

Figure R3-8. Minimal energy pathway in the transition process of the different three phases.

1. Zhang, L.; Yang, Z.; Gong, T.; Pan, R.; Wang, H.; Guo, Z.; Zhang, H.; Fu, X., Recent advances in emerging Janus two-dimensional materials: from fundamental physics to device applications. *J. Mater. Chem. A* **2020,** *8* (18), 8813-8830.
2. Urbankowski, P.; Anasori, B.; Makaryan, T.; Er, D.; Kota, S.; Walsh, P. L.;

Zhao, M.; Shenoy, V. B.; Barsoum, M. W.; Gogotsi, Y., Synthesis of two-dimensional titanium nitride Ti4N3 (MXene). *Nanoscale* **2016,** *8* (22), 11385-91.

3. Lu, A. Y.; Zhu, H.; Xiao, J.; Chuu, C. P.; Han, Y.; Chiu, M. H.; Cheng, C. C.; Yang, C. W.; Wei, K. H.; Yang, Y.; Wang, Y.; Sokaras, D.; Nordlund, D.; Yang, P.; Muller, D. A.; Chou, M. Y.; Zhang, X.; Li, L. J., Janus monolayers of transition metal dichalcogenides. *Nat Nanotechnol* **2017,** *12* (8), 744-749.

4. Zhang, J.; Jia, S.; Kholmanov, I.; Dong, L.; Er, D.; Chen, W.; Guo, H.; Jin, Z.; Shenoy, V. B.; Shi, L.; Lou, J., Janus Monolayer Transition-Metal Dichalcogenides. *ACS Nano* **2017,** *11* (8), 8192-8198.

5. Hill, N. A., Why Are There so Few Magnetic Ferroelectrics? *J. Phys. Chem. B* **2000,** *104*. 6. Feng, Y.; Zhang, T.; Dai, Y.; Huang, B.; Ma, Y., p-orbital multiferroics in singlelayer SiN. *Appl. Phys. Lett.* **2022,** *120* (19).

7. Peng, H.; Xiang, H. J.; Wei, S. H.; Li, S. S.; Xia, J. B.; Li, J., Origin and enhancement of hole-induced ferromagnetism in first-row d0 semiconductors. *Phys. Rev. Lett.* **2009,** *102* (1), 017201.

8. Pan, H.; Feng, Y. P.; Wu, Q. Y.; Huang, Z. G.; Lin, J., Magnetic properties of carbon doped CdS: A first-principles and Monte Carlo study. *Phys. Rev. B* **2008,** *77* (12), 125211.

9. Pan, H.; Yi, J. B.; Shen, L.; Wu, R. Q.; Yang, J. H.; Lin, J. Y.; Feng, Y. P.; Ding, J.; Van, L. H.; Yin, J. H., Room-temperature ferromagnetism in carbon-doped ZnO. *Phys. Rev. Lett.* **2007,** *99* (12), 127201.

10. Zhang, Q.; Zhang, Y.; Li, Y.; Fang, D.; Che, J.; Zhang, E.; Zhang, P.; Zhang, S., An intrinsic room-temperature half-metallic ferromagnet in a metal-free PN(2) monolayer. *Phys. Chem. Chem. Phys.* **2022,** *24* (11), 7077-7083.

11. Huang, C.; Feng, J.; Wu, F.; Ahmed, D.; Huang, B.; Xiang, H.; Deng, K.; Kan, E., Toward Intrinsic Room-Temperature Ferromagnetism in Two-Dimensional Semiconductors. *J. Am. Chem. Soc.* **2018,** *140* (36), 11519-11525.

12. J. Zhou, Q. W., Q. Sun, X. S. Chen, Y. Kawazoe, and P. Jena, Ferromagnetism in Semihydrogenated Graphene Sheet. *Nano Lett.* **2009,** *9*, 3867-3870.

13. Xie, X.; Chen, M.; Yu, Y.; Li, J.; Zhao, M.; Li, L., Structural phase transitions, mechanical properties, and electronic band structures of room-temperature ferromagnetic monolayers ScMP2 (M = Mn and Cr). *Results in Physics* **2023,** *53*.

14. Ai, H.; Li, F.; Bai, H.; Liu, D.; Lo, K. H.; Yang, S. A.; Kawazoe, Y.; Pan, H., Ferroelectricity coexisted with p-orbital ferromagnetism and metallicity in two-dimensional metal oxynitrides. *npj Computational Materials* **2022,** *8* (1).

15. Ding, W.; Zhu, J.; Wang, Z.; Gao, Y.; Xiao, D.; Gu, Y.; Zhang, Z.; Zhu, W., Prediction of intrinsic two-dimensional ferroelectrics in In2Se3 and other III2-VI3 van der Waals materials. *Nat Commun* **2017,** *8*, 14956.

jz-2023-03527c.R2

Name: Peer Review Information for "Two-dimensional Asymmetric Multiferroics: Unique Way Toward Strong Magnetoelectric Coupling and Multi-State Memory"

Second Round of Reviewer Comments

Reviewer: 3

Comments to the Author

The authors addressed my concerns in the present version. I am satisfied with the reversion and responses, and thus recommend publication in JPCL.

Reviewer: 1

Comments to the Author

The auhtors have addressed my concerns and I recommend it for publication.

Author's Response to Peer Review Comments:

Hui Pan, Professor
Institute of Applied Physics and Materials Engineering
N23-4003, University of Macau, Macao S.A.R., China
Office Tel: (853) 88224427
E-mail: huipan@um.edu.mo

February 1, 2024

Manuscript ID: jz-2023-03527c.R1

Dear Dr. Editor,

Thank you very much to accept our manuscript entitled “Two-dimensional Asymmetric Multiferroics: Unique Way Toward Strong Magnetoelectric Coupling and Multi-State Memory” (Manuscript ID: jz-2023-03527c.R1). All the comments are helpful to improve our work and guide our future research. We had finished the non-scientific changes requested and summarized them below. We expect that the revised manuscript can satisfy the requirements and meet the high standards for publication in The Journal of Physical Chemistry Letters. If you have any concern, please do not hesitate to contact me.

Summary of Changes:

1. The line numbering from manuscript file was removed.
2. The TOC graphic was resized to 2 in × 2 in and moved to the correct position.
3. The section headings throughout the body of the manuscript were removed.

Yours sincerely,

Prof. Hui Pan, PhD
